# The polarity protein Par3 coordinates positively self-renewal and negatively invasiveness in glioblastoma

**DOI:** 10.1038/s41419-021-04220-7

**Published:** 2021-10-12

**Authors:** Mahsa Shahidi Dadras, Laia Caja, Artur Mezheyeuski, Sijia Liu, Caroline Gélabert, Maria Catalina Gomez-Puerto, Radiosa Gallini, Carl-Johan Rubin, Peter ten Dijke, Carl-Henrik Heldin, Aristidis Moustakas

**Affiliations:** 1 Department of Medical Biochemistry and Microbiology, Science for Life Laboratory, Box 582, Biomedical Center, Uppsala University, SE-75123 Uppsala, Sweden; 2 Department of Immunology, Genetics and Pathology, Rudbeck Laboratory, Science for Life Laboratory, Uppsala University, SE-75185 Uppsala, Sweden; 3 Brain and Mind Research Institute, Weill Cornell Medicine, New York, NY 10021 USA; 4 Department of Cell and Chemical Biology, Oncode Institute, Leiden University Medical Center, Leiden, The Netherlands

**Keywords:** Cell polarity, CNS cancer

## Abstract

Glioblastoma (GBM) is a brain malignancy characterized by invasiveness to the surrounding brain tissue and by stem-like cells, which propagate the tumor and may also regulate invasiveness. During brain development, polarity proteins, such as Par3, regulate asymmetric cell division of neuro-glial progenitors and neurite motility. We, therefore, studied the role of the Par3 protein (encoded by *PARD3*) in GBM. GBM patient transcriptomic data and patient-derived culture analysis indicated diverse levels of expression of *PARD3* across and independent from subtypes. Multiplex immunolocalization in GBM tumors identified Par3 protein enrichment in SOX2-, CD133-, and NESTIN-positive (stem-like) cells. Analysis of GBM cultures of the three subtypes (proneural, classical, mesenchymal), revealed decreased gliomasphere forming capacity and enhanced invasiveness upon silencing Par3. GBM cultures with suppressed Par3 showed low expression of stemness (*SOX2* and *NESTIN*) but higher expression of differentiation (*GFAP*) genes. Moreover, Par3 silencing reduced the expression of a set of genes encoding mitochondrial enzymes that generate ATP. Accordingly, silencing Par3 reduced ATP production and concomitantly increased reactive oxygen species. The latter was required for the enhanced migration observed upon silencing of Par3 as anti-oxidants blocked the enhanced migration. These findings support the notion that Par3 exerts homeostatic redox control, which could limit the tumor cell-derived pool of oxygen radicals, and thereby the tumorigenicity of GBM.

## Introduction

Glioblastoma (GBM) is a lethal tumor of the central nervous system, whose patients survive approximately 12−14 months after diagnosis [1, 2]. GBM treatment involves surgical resection followed by radiotherapy and adjuvant chemotherapy with temozolomide [3, 4]. Genomic/transcriptomic analyses classify GBM according to genetic mutations, gene copy number changes, and gene expression profiles, in proneural (PN), classical (CL) and mesenchymal (MS) tumors [5, 6]. A subclass of cells within the GBM tumor mass, called glioblastoma stem-like cells, are thought to initiate and maintain tumor growth and resemble adult brain stem cells [7, 8]. The GBM stem-like cells self-renew and propagate heterogeneous tumors upon transplantation to recipient animal brains [7, 8].

Dynamic changes in adhesion, polarity, and cyto-architecture are implicated in tumor growth, invasion and metastasis [9, 10]. Polarity proteins regulate apical-basal polarity in epithelial and endothelial cells, and dendrite-axon polarity in differentiating neurons, including directionality of cell migration and directed vesicular transport; indirectly, these proteins also control cell proliferation and survival [10, 11]. Polarity proteins form a multiprotein complex comprising Par3, Par6, atypical protein kinase C, and downstream small GTPases. The Par complex guides assembly of epithelial or neuronal cell−cell junctions, leading to compartmentalization of the plasma membrane, and specifies axonal differentiation in neural/glial progenitor cells [12, 13]. In radial glial progenitors, Par3 interacts with the protein Numb, which regulates the activity of the Notch receptor, thus ensuring asymmetric division that generates one progenitor cell and one cell that differentiates to a neuron [14]. By interacting with ASPP2 (apoptosis stimulating proteins of p53), Par3 promotes tight junction integrity in early neural progenitor cells [15], whereas dephosphorylation of Par3 by Smec/protein phosphatase-4, inhibits Par3 function and promotes neuronal differentiation [16]. In addition to being a unit of the polarity complex, Par3 oligomerizes and associates with microtubules, inducing their bundling and neuronal polarization [17]. Interaction between Par3, Par6, and the membrane protein NGL2 (netrin-G ligand-2), stabilizes microtubular bundles at the axonal tip during neurite differentiation [18].

Par3 can act as a tumor suppressor in epithelial cancers [10, 19]. In GBM, deletions of exons 3 to 20 or exon 25 of the *PARD3* gene have been identified, causing disruption of tight junctions in astrocytes; reconstitution of Par3 expression in defective GBM cell lines restored tight junction formation [20]. Since little is known about the relevance of polarity proteins in GBM biology [19], we studied the significance of Par3 in gliomasphere formation and invasiveness. Unexpectedly, our results show that Par3 has dual functions in GBM cell biology; decreasing Par3 expression enhanced invasion by increasing oxidative stress, while it impaired gliomasphere forming potential and ATP production.

## Results

### *PARD3* is expressed in GBM patients and patient-derived cell cultures of all three subtypes

**Fig. 1:**
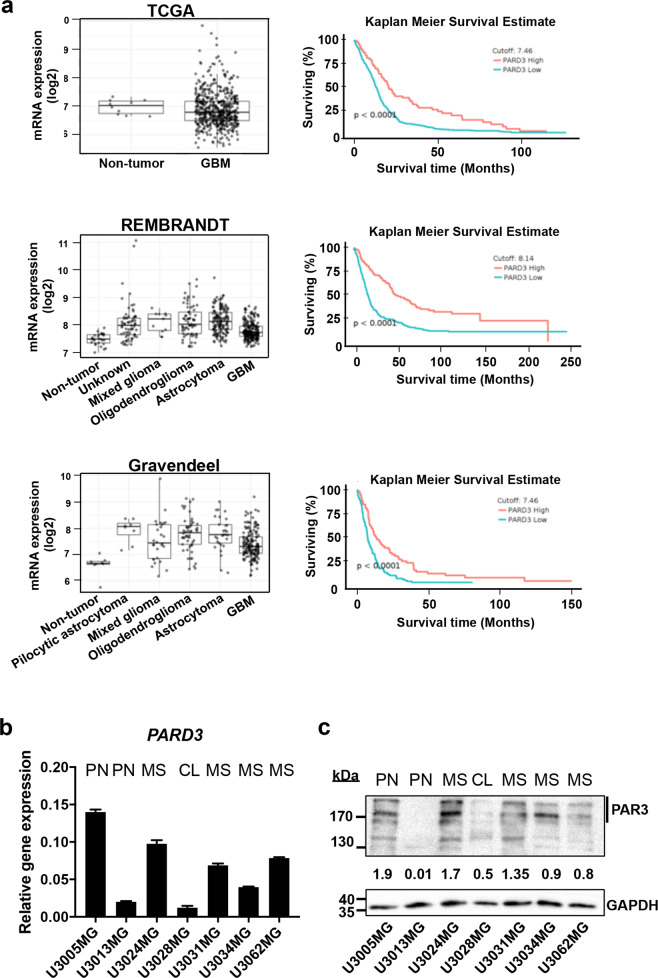
Expression of Par3 in glioblastoma. **a** Expression levels of *PARD3* mRNA (left panels) in GBM samples compared to non-tumoral tissue only (TCGA dataset), or to non-tumoral and other brain malignancy tissues (REMBRANDT, Gravendeel datasets). Kaplan Meier survival curves of GBM patients with low (blue curves) and high (red curves) levels of *PARD3* mRNA expression according to the three datasets (TCGA, REMBRANDT, and Gravendeel) obtained using the GlioVis data portal. Statistical significance of the difference between the two curves is also indicated. **b** *PARD3* mRNA expression relative to *GAPDH* mRNA in patient-derived GBM cultures (PN proneural; CL classical; MS mesenchymal subtype); results are expressed as mean ± SEM (*n* = 2, technical triplicate). **c** Par3 protein expression level in the same panel of cultures; GAPDH is used as a total protein loading control with densitometric values of Par3 relative to GAPDH listed and molecular size markers in kDa shown.

We analyzed *PARD3* gene expression in GBM versus other glioma types and non-tumor tissues in The Cancer Genome Atlas (TCGA), Gravendeel, and REMBRANDT databases [21–23] using the GlioVis data portal [24]. *PARD3* expression was rather diverse in GBM samples when compared to non-tumor tissue, including patients with very high *PARD3* levels and others with rather low levels; yet, the median value of expression appeared lower in GBM relative to non-tumoral samples (considering, however, the limited number of non-tumoral samples included in TCGA) (Fig. 1a). The REMBRANDT and Gravendeel datasets, which enlist fewer GBM cases than TCGA, showed a larger variation in *PARD3* expression levels and these datasets demonstrated a median *PARD3* expression that was higher in GBM relative to non-tumoral samples (Fig. 1a). Consistently among the three datasets, low *PARD3* levels correlated with poorer patient survival (Fig. 1a).

Analyzing seven patient-derived cultures from the Human Glioblastoma Cell Culture (HGCC) resource [25], demonstrated a group of 5 cultures with detectable (U3005MG/PN, U3024MG/MS, U3031MG/MS, U3034MG/MS, U3062MG/MS) and two cultures with low/undetectable (U3013MG/PN, U3028MG/CL) Par3 mRNA and protein levels (Fig. 1b, c). By sequencing all 25 exons and flanking intronic sequences of the human *PARD3* gene in each of the seven cultures, we identified one single nucleotide polymorphism (SNP) in exon-7 (His^287^, synonymous change) and one in exon-25, in the 3′ untranslated region, represented in every culture (common SNPs, Supplementary Fig. S1a). Five SNPs scored only in specific cultures (unique SNPs, Supplementary Fig. S1a), corresponding to flanking intronic or exonic sequences, the latter resulting in synonymous alterations. We concluded that the seven GBM cultures carry wild-type *PARD3* and the identified SNPs may not have a functional impact. The SNP analysis revealed mainly homozygosity and, in some cases, hemizygosity (not shown). The HGCC resource reports genome-wide gene copy number data for all cell cultures [25]. The *PARD3* locus on chromosome 10p11.22-p11.21 is represented in HGCC by three genes, *NRP1* and *CREM* that flank *PARD3* and *PTEN* farther away (Supplementary Fig. S1b). All GBM cultures (except U3005MG) suffered reduction in copy number of the extended genomic locus (Supplementary Fig. S1b). However, the degree of gene copy loss (Supplementary Fig. S1b) did not strictly correlate with the level of Par3 protein expression (Fig. 1c), suggesting that Par3 protein levels are controlled either transcriptionally (Fig. 1b) or post-transcriptionally.

### Depleting Par3 decreases gliomasphere formation

**Fig. 2:**
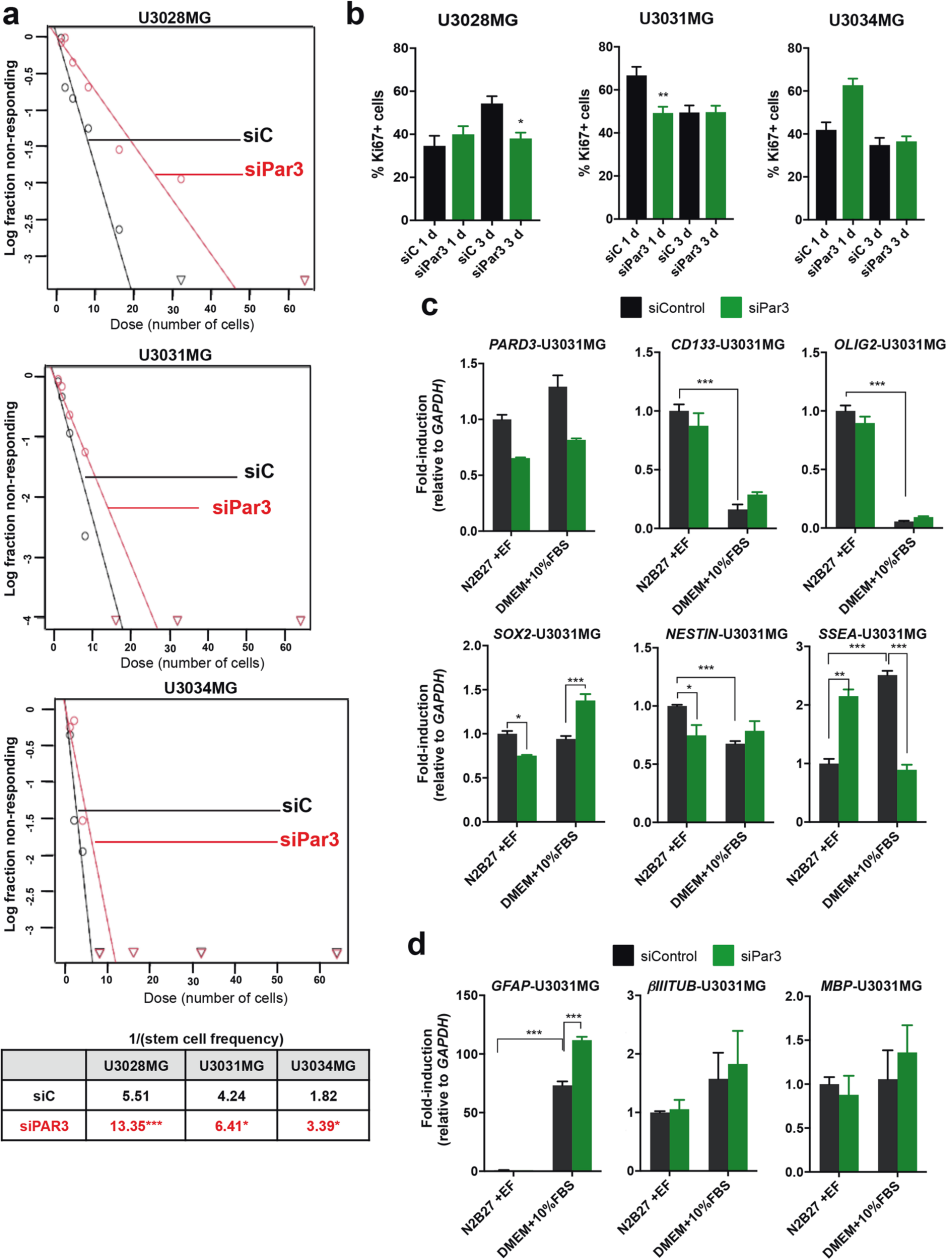
Reduction in Par3 depletes gliomasphere formation. U3028MG, U3031MG, and U3034MG cells were transfected with control and Par3 siRNA. **a** ELDA expressing median values from transfected cells (siControl, black curves; siPar3, red curves). High *x*-axis intercept corresponds to low number of gliomaspheres; note the large degree of shift of the median curves to the right upon Par3 silencing. The table shows the stem cell frequency (1 stem cell/*x* cells). For U3028MG and U3034MG: *n* = 2 with seven replicates, and for U3031MG: *n* = 4 with seven replicates. **b** Percentage of Ki67-positive cells in U3028MG, U3031MG and U3034MG cultures. Average measurements of *n* = 2 (U3028MG), *n* = 3 (U3031MG), *n* = 5 (U3034MG) independent experiments, 10−15 pictures analyzed per experiment, and associated SEM are plotted (siControl, black bars; siPar3 green bars). Statistical comparison (one-way Anova) indicates significant differences, **p < 0.01, ***p < 0.001. **c**, **d** Expression of the indicated genes in cells cultured in N2B27 plus EGF/FGF2 (N2B27 + EF, stem cell medium) or DMEM/10% FBS after 5 days of treatment; results are mean ± S.E.M. of two independent experiments with technical triplicates. Statistical comparison (one-way Anova); significant differences, *p < 0.05, ***p < 0.001.

We analyzed the functional role of Par3 in U3005MG/PN, U3028MG/CL, U3034MG/MS, and U3031MG/MS cultures in order to cover all three major GBM subtypes, and selected cells that expressed high Par3 levels (however, the single CL cell culture U3028MG/CL expressed rather low Par3 levels as explained above; Fig. 1c). The cells were cultured as non-adherent spheroids [26], and endogenous Par3 was silenced using a pool or four individual short interfering RNAs (siRNAs); the pool, and at least two individual siRNAs demonstrated silencing efficiency of 50−95% depending on the culture and the time period after transfection (Supplementary Fig. S2a−c). We chose to genetically perturb endogenous Par3 without attempting to overexpress exogenous Par3 into GBM cultures with low endogenous levels, as our previous experience with tumor suppressor proteins had indicated that transfected cells die when cultured under low-adhesion conditions. Thus, Par3 reduction caused a highly reproducible decrease in gliomasphere-forming frequency in all four cultures, as measured by extreme limiting dilution assays (ELDA; Fig. 2a, Supplementary Fig. S2d).

Ki67 immunostaining showed decreased proliferation after Par3 silencing at specific time points in some of the GBM cultures (Fig. 2b). Cell viability (MTS) assays also showed a weak reduction in viability at specific time points after Par3 silencing in some of the cultures (Supplementary Fig. S3a). Corroborating these results, no significant change in apoptotic cell numbers was observed, monitored either by annexin-V/PI staining (data not shown) or caspase-3 activity, assayed enzymatically or by immunoblot analysis (Supplementary Fig. S3b, c). Since autophagy can support cancer stem cell viability [27], we analyzed autophagic vacuole accumulation by flow cytometry (Supplementary Fig. S3d). A small but significant decrease in the amount of autophagic vacuoles was evident in U3031MG/MS but not in U3034MG/MS after silencing Par3 (Supplementary Fig S3d). Thus, the robust effects of Par3 silencing on gliomasphere formation could not be accounted for by strong effects on cell proliferation/viability, apoptosis, or autophagic flux.

The effect of Par3 silencing on gliomasphere formation (Fig. 2a and Supplementary Fig. S2d) could be due either to reduced expression of stem cell genes or to increased expression of differentiation genes. We measured mRNA expression of established stem cell-like markers in GBM, the pentaspanning glycoprotein *CD133* (*AC133* or *prominin-1*), the transcription factors *OLIG2* and *SOX2* (*sex-determining region Y-box 2*), the intermediate filament *NESTIN*, the embryonic glycoprotein *SSEA-1* (*stage-specific embryonic antigen-1*), the astrocytic differentiation and intermediate filament gene *GFAP* (*glial fibrillary acidic protein*), the neuronal marker *βIIITub* (*βIII-TUBULIN*) and the Schwann cell lineage marker *MBP* (*myelin basic protein*) in U3031MG/MS (Fig. 2c, d). Par3 silencing did not significantly affect the basal expression of *CD133* or *OLIG2* but caused a decrease in the levels of *SOX2* and *NESTIN* and an increase in *SSEA-1* expression, under stem-like cell culture conditions (Fig. 2c; N2B27 + EF). No effect either was observed on the very low basal expression of *GFAP* or on *βIIITub* and *MBP* expression under the same conditions (Fig. 2d). Switching the GBM culture to conditions that enrich for astrocytic differentiation marker expression (DMEM + 10% fetal bovine serum (FBS)), resulted in a dramatic reduction of *CD133*, *OLIG2* and smaller but significant reduction in *NESTIN* expression, increase in *SSEA-1* and *GFAP*, but no effect on *SOX2*, *βIIITub* and *MBP* levels (Fig. 2c, d). These gene expression changes attested that the GBM cultures, although tumor cells, maintained a relative ability to adapt under conditions that promote astrocytic differentiation of normal glial progenitors, by decreasing expression of several (but not all) stem cell genes and increasing strong expression of GFAP (Fig. 2c, d). Under DMEM + 10% FBS conditions, Par3 silencing significantly increased *SOX2*, *GFAP*, and reduced *SSEA-1* expression (Fig. 2c, d). The gene expression assays suggest that endogenous Par3 has a mild impact on specific stem cell-like and differentiation genes, but the mechanism of gliomasphere suppression upon its silencing may rely on additional mechanisms.

### Par3 inhibits GBM migration-invasion

**Fig. 3:**
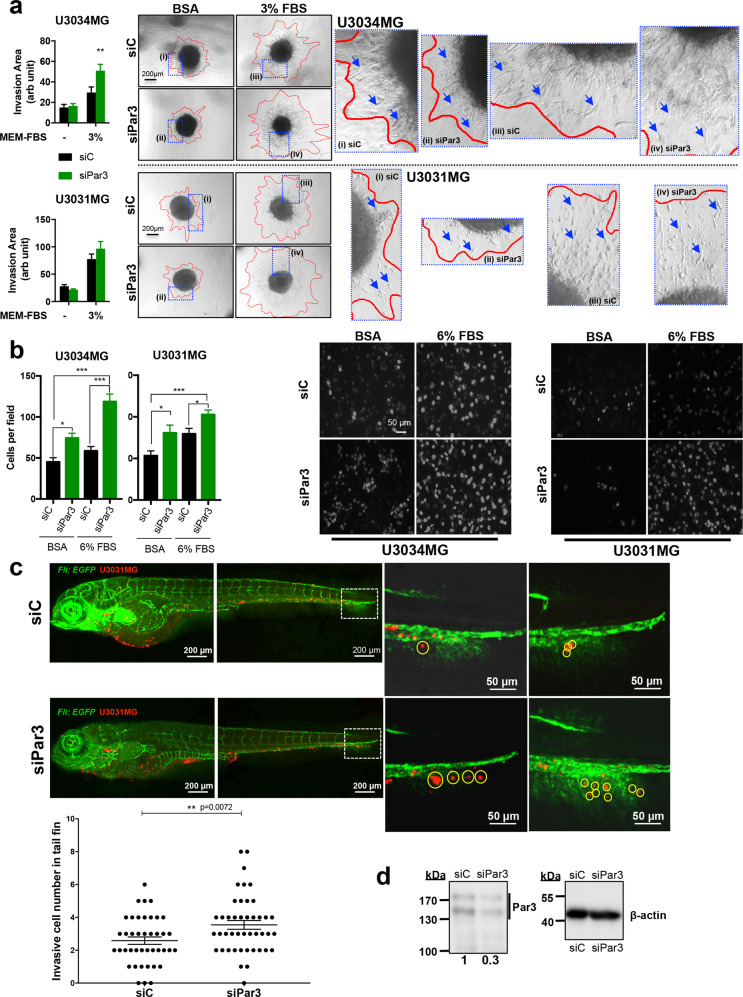
Reduction in Par3 enhances cell migration and invasion. **a** Spheroids of U3031MG and U3034MG transfected with control (siC, black bars) or Par3 (green bars) siRNA embedded in collagen, invaded the matrix in the presence of MEM containing BSA (-) or MEM containing 3% FBS, and assayed after 48 h. Quantification (left) expresses results as mean ± SEM out of multiple independent experiments (for U3031MG, *n* = 2 with five biological replicates; for U3034MG, *n* = 4 with 3 biological replicates), and statistical comparison (t-test) indicates significant differences, *p < 0.05, **p < 0.01. Representative photomicrographs with red lines demarcating the outer rim formed by invasive cells (right); blue rectangles indicate magnified areas of the invasive rim with arrows pointing to invading cells. **b** Transwell-based invasion assays of U3031MG and U3034MG cells transfected with control (siC, black bars) or Par3 (green bars) siRNA, migrating through laminin towards DMEM/BSA or DMEM/6% FBS. Left, quantification of the number of cells per field (for U3034MG, *n* = 2 and for U3031MG, *n* = 3, in duplicate each time; for each independent experiment 15 different fields were quantified). Right, representative photomicrographs of stained nuclei of invasive cells (magnification bar, 50 µm). Results are expressed as mean ± SEM and statistical comparison (t-test) indicates significant differences, *p < 0.05, ***p < 0.001. **c** Invasive capacity of CM-Dil-labeled U3031MG cells after silencing Par3 was assessed in vivo after injecting approximately 400 cells in the transgenic *Fli:EGFP* zebrafish embryos with endothelial-specific EGFP expression. Extravasation and collagenous tail fin invasion were observed. One representative image of the whole zebrafish from each group is shown in the left panel, with two zoom-in images of the invasive cells in the tail fins of two different zebrafish from each group shown on the right panel. Invasive cells are indicated with yellow circles in the zoom-in images. Quantification of invading U3031MG numbers per fish, *n* = 40, presented as MEM ± S.E.M (left). Statistical comparison (t-test); significant differences, **p < 0.01. **d** Immunoblot demonstrating Par3 silencing efficiency in U3031MG cells injected in the zebrafish embryos. β-Actin serves as loading control, densitometric values of Par3 expression relative to β-actin are listed and molecular size markers in kDa are shown.

Previous studies have highlighted the importance of loss in cell polarity for tumor invasion and metastasis [9, 10, 28]. GBM cell invasion into collagen type I matrix out of collagen-embedded gliomaspheres was stimulated by switching the culture medium to 3% FBS (Fig. 3a). Following Par3 silencing, U3034MG/MS cultures demonstrated elevated invasion rates, and U3031MG/MS cells showed the same trend (Fig. 3a). All invading cells appeared as either solitary or small groups of extremely elongated cells (Fig. 3a, arrows). Invasiveness through a laminin matrix, a more physiological (relative to collagen type I) brain matrix protein, in a transwell-based assay, was enhanced upon Par3 silencing in U3031MG/MS, U3034MG/MS, and U3005MG/PN cells transfected either with the siRNA pool (Fig. 3b) or individual siRNAs (Supplementary Fig. S4a). Performing the invasion assay in U3028MG/CL cells did not generate significant effects, probably due to the lower basal rate of invasion of these cells (Supplementary Fig. S4b). It should be noted that U3028MG/CL cells express low Par3 protein levels (Fig. 1c).

Invasiveness in vivo was further assessed by injecting the transfected CM-Dil Dye-labeled U3031MG/MS cells in the duct of Cuvier of transgenic Tg(Fli1::EGFP/enhanced green fluorescent protein) zebrafish embryos (Fig. 3c). In this transgenic line, the endothelial *Fli1* gene promoter drives the expression of EGFP in blood vessels. Extravasating CM-Dil/U3031MG/MS cells invaded the collagenous matrix of the tail (Fig. 3c; magnified insets with circled extravasated cells). After Par3 silencing (Fig. 3d), higher numbers of CM-Dil/U3031MG/MS cells invaded the fish tail (Fig. 3c). The in vitro and in vivo results suggest that endogenous Par3 limits migratory/invasive potential in GBM cells.

### GBM transcriptomic analysis points to the regulation of oxidative phosphorylation

**Fig. 4:**
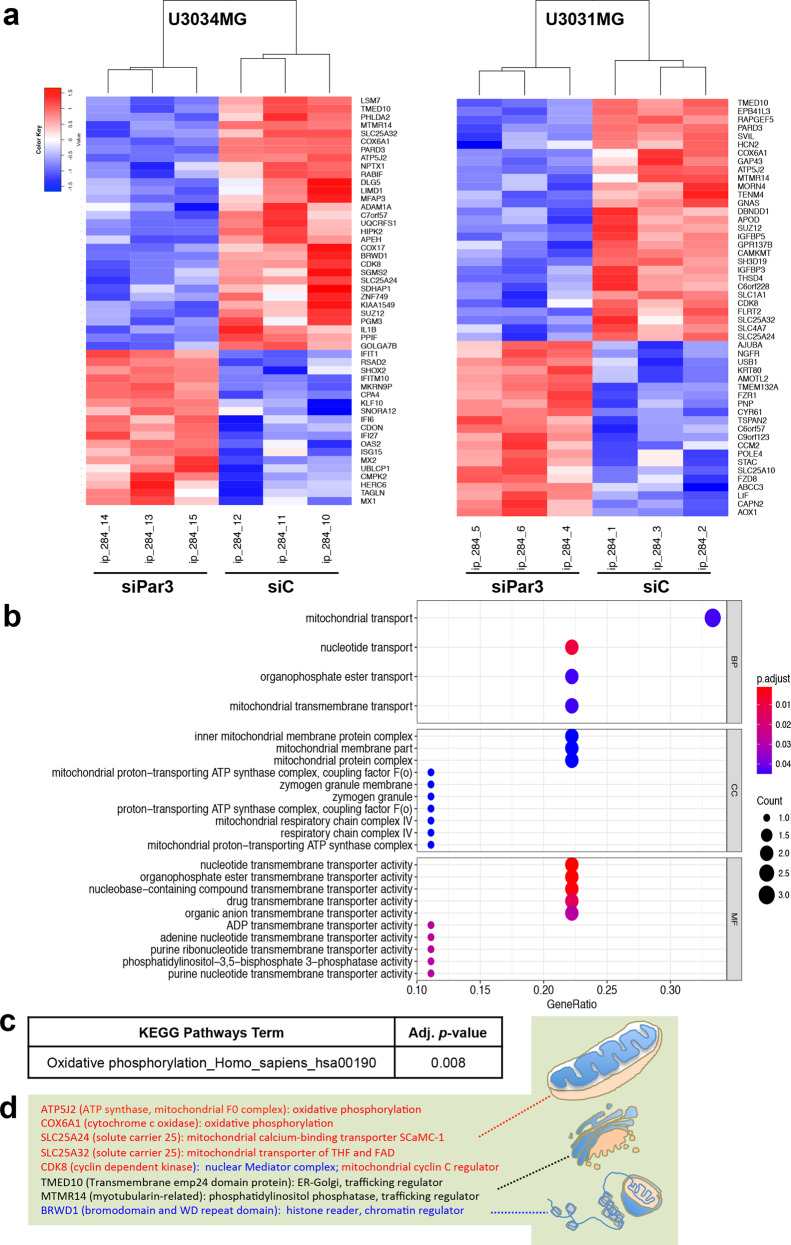
Transcriptomic analysis of U3031MG and U3034MG cells. **a** Heatmap of the top-50 regulated genes (adjusted *p*-value < 0.05) after Par3 silencing (siPar3 vs siC) in U3034MG and U3031MG cultures. Fold-change in expression is color-coded (high, red; low, blue) in triplicate samples per biological condition. **b** Gene Ontology enrichment for Biological Process (BP) of the common down-regulated genes after Par3 silencing. The adjusted *p*-value is color-coded and the number of genes per category is shown by circle diameter. **c** KEGG pathway analysis for downregulated genes after Par3 silencing with the corresponding adjusted *p*-value. **d** A list of common downregulated genes after Par3 silencing along with their known functions represented schematically (mitochondria, endoplasmic reticulum-Golgi apparatus, and chromatin).

We explored further actions of Par3 in GBM by transcriptomic analysis after Par3 silencing in U3031MG/MS and U3034MG/MS cells (Fig. 4). We analyzed the two mesenchymal cell cultures that exhibited reproducible results in the above phenotypic analysis, without including the proneural and classical cell models, in order to minimize differences caused by GBM subtype and not by Par3 silencing. Reduction in Par3 led to cell adaptation characterized by significant numbers of differentially expressed genes defined based on an adjusted *p*-value < 0.05 (Fig. 4a). In total, 173 genes changed expression in U3031MG/MS cells (93 downregulated, 80 upregulated) and 36 genes in U3034MG/MS cells (22 downregulated, 14 upregulated). The heat-maps of the two GBM cultures were rather similar, yet the specific differentially expressed genes were rather unique in each GBM culture, possibly reflecting their origin from independent patients. Accordingly, 8 downregulated genes were identified in both GBM cultures. Gene Ontology analysis of these downregulated genes using the gene set enrichment analysis web Enrichr tool [29], indicated Biological Processes related to mitochondrial transport, folic acid transport and metabolism, regulation of cell death, and ATP biosynthesis (Fig. 4b). KEGG pathway analysis further indicated that the downregulated genes belong to pathways of oxidative phosphorylation (Fig. 4c). Specifically, we identified two enzymes of the oxidative phosphorylation (ATP5J2, COX6A1), two mitochondrial transporters (SLC25A24, SLC25A32), a protein kinase active in the nucleus and in mitochondria (CDK8), two trafficking regulators (TMED10, MTMR14) and a chromatin regulator (BRWD1) (Fig. 4d). This information prompted us to assess the mitochondrial function in the GBM cells under conditions of Par3 silencing.

### Reduction in Par3 disrupts GBM mitochondrial ATP production and induces ROS

**Fig. 5:**
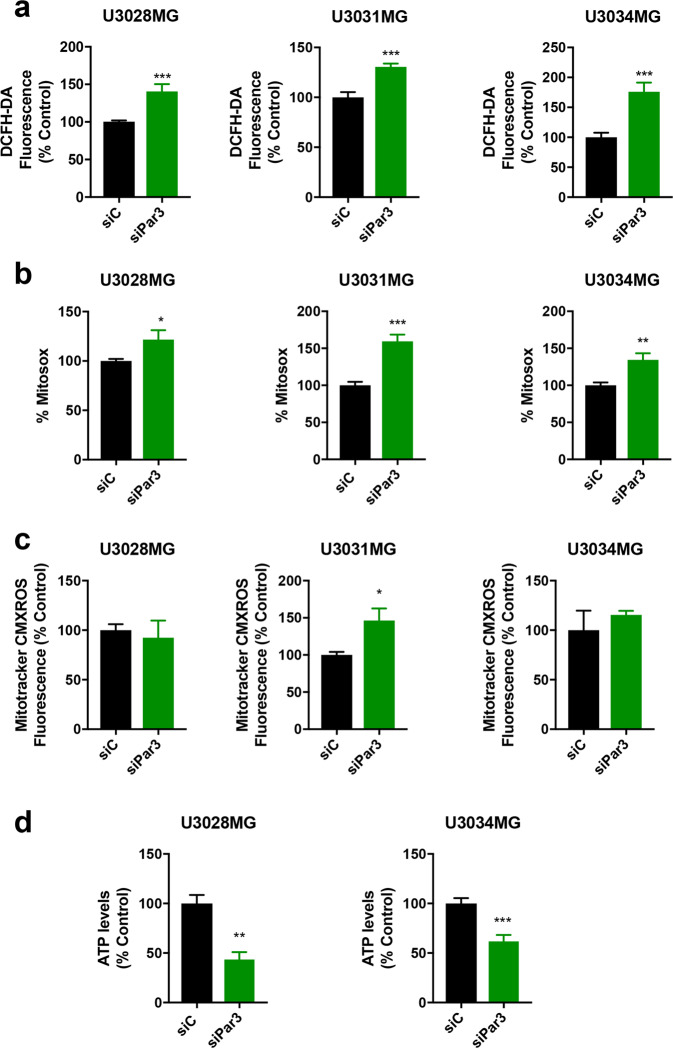
Par3 regulates ROS and mitochondrial function in GBM cells. U3028MG, U3031MG, and U3034MG cultures were transfected with control (siC, black bars) and Par3 (green bars) siRNAs. **a** Intracellular ROS content measured by DCFH-DA fluorescence is expressed as percent of the control siRNA (siC) on each day. For U3028MG, *n* = 2 in four duplicates for day 1, *n* = 2 in two duplicates for day 3; for U3031MG, *n* = 2 in four duplicates for days 1 and 3; for U3034MG, *n* = 3 in four duplicates for day 1, *n* = 2 in four duplicates for day 3. **b** Mitochondrial ROS measured by MitoSOX-Red is expressed as percent of the control siRNA (siC). For U3031MG and U3034MG, *n* = 5 in triplicate; for U3028MG, *n* = 3 in triplicate. **c** Mitochondrial transmembrane potential analysis using MitoTracker CMXROS at day 3. For U3034MG: *n* = 4; for U3028MG and U3031MG, *n* = 2. **d** ATP levels in U3028MG and U3034MG cells expressed as percent of the control siRNA (siC). For U3028MG, *n* = 2; for U3034MG, *n* = 4. All results are expressed as mean ± SEM of at least two repeats; statistical comparison (t-test) indicates significant differences, *p < 0.05, **p < 0.01, **p < 0.001.

Mitochondrial disruption can induce oxidative stress. Elevated levels of reactive oxygen species (ROS) in various tumor cells are thought to be tumorigenic [30, 31]. We measured endogenous ROS levels after silencing Par3 either by siRNA pool (Fig. 5) or by individual siRNAs (Supplementary Fig. S5), in U3005MG/PN, U3028MG/CL, U3031MG/MS, and U3034MG/MS cultures. All cultures showed significantly elevated levels of intracellular ROS measured by 2′, 7′-dichloro-dihydro-fluorescein diacetate (DCFH-DA; Fig. 5a, Supplementary Fig. S5a), which coincided with an increase in mitochondrial superoxide, measured by MitoSOX-Red (Fig. 5b). Extracellular ROS did not change significantly after Par3 reduction (Supplementary Fig. S5b). The increase in mitochondrial ROS did not lead to disruption of mitochondrial membrane potential, measured by MitoTracker CMXROS (Fig. 5c) while resulting in a significant (50%) reduction of ATP levels (Fig. 5d).

Par3 silencing did not affect GBM cell mitochondrial mass, as measured by MitoTracker Deep Red analysis (Supplementary Fig. S5c). We quantified mitochondrial branch length and the total mitochondrial footprint using structured illumination super-resolution microscopy after incubation of cells with MitoTracker Deep Red; no changes caused by Par3 silencing could be recorded (SupplementaryFig. S5d). These results suggest that Par3 may have an impact on mitochondrial function, rather than on mitochondrial mass or architecture.

### Antioxidants rescue the impact of Par3 reduction on GBM cell invasion

**Fig. 6:**
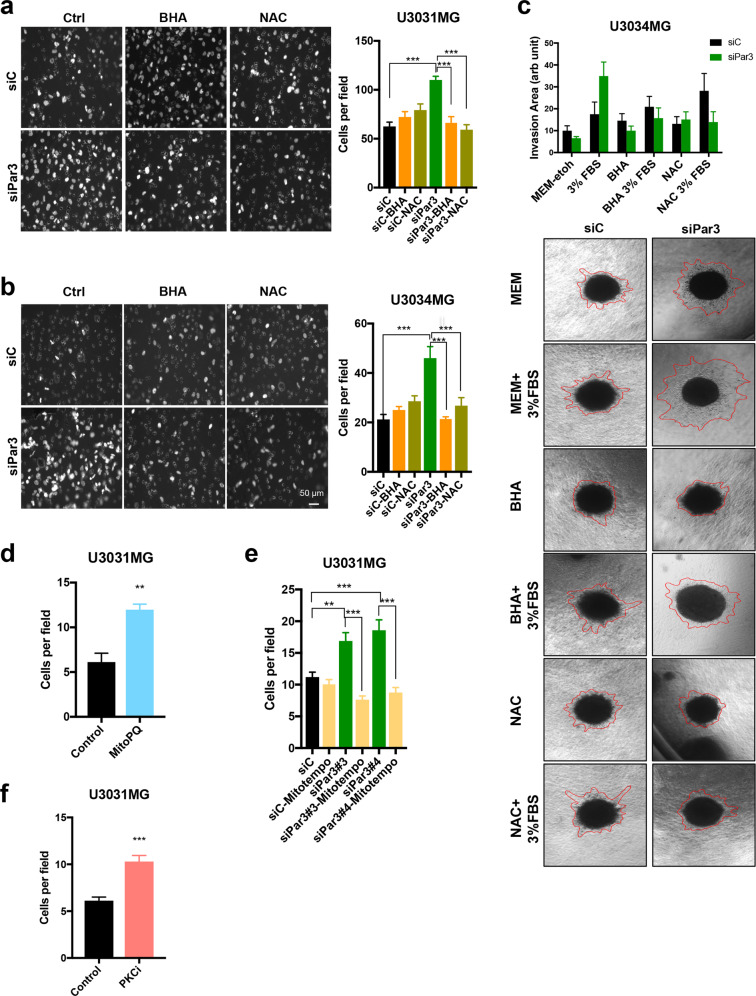
Antioxidants counteract the enhanced invasion caused by Par3 silencing. **a, b** Transwell-based invasion assay of transfected U3031MG and U3034MG cells migrating through laminin towards DMEM/6% FBS in the absence (Ctrl) or presence of antioxidants BHA (200 µM) or NAC (5 mM). Representative images of invasive cells (left); magnification bar, 50 µm. Quantification of the number of cells per field (right, *n* = 2 in duplicate, 15 different fields per independent experiment, and results are expressed as mean ± SEM). **c** Spheroids embedded in collagen were treated with antioxidants BHA (200 µM) and NAC (5 mM) in the presence of MEM or MEM/3% FBS, and representative photomicrographs were recorded after 48 h. Red lines demarcate the outer rim formed by invasive cells. Quantification of the invasion area (top). Results are expressed as mean ± SEM of three replicates of *n* = 2 independent experiments; siControl (siC), black bars; siPar3, green bars. Transwell-based invasion assay of U3031MG cells through laminin towards DMEM/6% FBS without (Ctrl) or with 100 µM MitoPQ (**d**) or 1 µM aPKCi (**f**). The number of invading cells per field was quantified (*n* = 2 in duplicate, 10 different fields per independent experiment were quantified; results are expressed as mean ± SEM). **e** Transwell-based invasion assay of U3031MG cells transfected with control or Par3 siRNAs through laminin towards DMEM/6% FBS in the absence or presence of 50 µM MitoTEMPO; analyses were performed as above.

We have so far observed that Par3 reduction pre-disposed GBM cells to increased invasion and production of ROS (Figs. 3 and 5). In order to investigate whether ROS play any role in GBM cell invasion under the influence of Par3, we assessed invasiveness of U3031MG/MS and U3034MG/MS cells upon silencing of Par3 and further treatment with two antioxidants, butyl-hydroxyanisole (BHA) and N-acetyl-L-cysteine (NAC), in the transwell assay using a laminin matrix (Fig. 6). Treatment with BHA and NAC had only minimal and not significant effects on GBM cell invasion (Fig. 6a, b). However, once invasiveness was induced by serum in cells with Par3 silencing, the two antioxidants reduced invasion down to basal levels (Fig. 6a, b). Moreover, when collagen-embedded gliomaspheres were treated with BHA, invasion stimulated by serum, and enhanced after Par3 silencing, was inhibited (Fig. 6c). Under the same conditions, treatment with NAC by itself did not affect invasiveness; however, upon serum-stimulation, NAC appeared to enhance invasiveness, but still blocked invasion induced after Par3 silencing (Fig. 6c).

Superoxide is a mitochondrial ROS with a major role in pathological oxidative stress and redox signaling. Mitochondria-targeted Paraquat (MitoPQ) enables the selective generation of superoxide within mitochondria [32]. As predicted from the previous experiments, MitoPQ facilitated the U3031MG/MS invasion induced by serum in the transwell-laminin assay (Fig. 6d). To investigate more specifically whether mitochondrial oxidative stress is critical for GBM invasiveness, we assessed invasion of U3031MG/MS cells in the transwell-laminin assay, by combining Par3 silencing with MitoTEMPO treatment, a mitochondria-targeted antioxidant (Fig. 6e). MitoTEMPO treatment reduced U3031MG/MS invasiveness induced by serum after Par3 silencing, to basal levels (Fig. 6e).

In the absence of specific chemical inhibitors targeting Par3, we used an inhibitor against aPKC (aPKCi), the catalytic Par3 partner (Fig. 6f). The aPKCi enhanced significantly the invasive capacity of U3031MG/MS (Fig. 6f), similar to the effect of Par3 silencing (Figs. 3b and 6a, e).

### Elevated ROS levels phenocopy Par3 silencing by suppressing gliomasphere formation

**Fig. 7:**
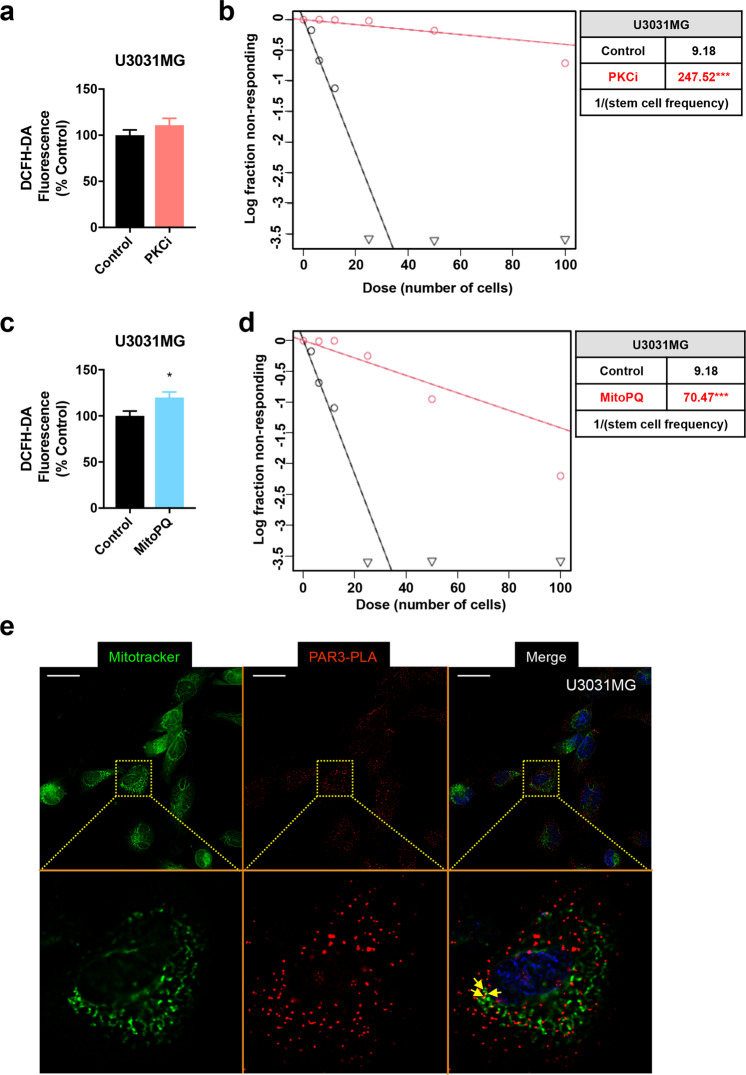
Inhibiting aPKC or inducing mitochondrial ROS disrupts gliomasphere formation. **a** Intracellular ROS content measured by DCFH-DA fluorescence after treatment of U3031MG cells with 1 µM aPKCi and expressed as a percent of control after 3 days. **b** ELDA expressing median values from U3031MG treated with 1 µM aPKCi for 10 days (Control, black curves; aPKCi treated, red curves). Note the large degree of shift of the median curves to the right upon aPKCi treatment. The table shows the stem cell frequency (1 stem cell/*x* cells); *n* = 3 with six replicates. **c** Intracellular ROS content measured by DCFH-DA fluorescence after treatment of U3031MG cells with 100 µM MitoPQ for 3 days and expressed as a percent of control. **d** ELDA expressing median values from U3031MG treated with 100 µM MitoPQ for 10 days (Control, black curves; MitoPQ treated, red curves). Note the large degree of shift of the median curves to the right upon MitoPQ treatment. The table shows the stem cell frequency (1 stem cell/*x* cells); *n* = 3 with six replicates. **e** Par3 localization in proximity to mitochondrial networks revealed by in situ PLA. MitoTracker Deep Red staining of mitochondria is represented in green, Par3 molecules are represented as single red dots and nuclei are visible in blue. Insets (dotted rectangles) magnify a single cell for a better visual effect. Arrows indicate co-localization. Magnification bars, 10 µm.

Encouraged by the impact of aPKCi on GBM cell invasion (Fig. 6f), we analyzed the impact of this inhibitor on intracellular ROS generation (measured by DCFH-DA fluorescence) in the same U3031MG/MS cells (Fig. 7a). ROS levels were not affected by the aPKCi (Fig. 7a). However, the aPKCi significantly decreased U3031MG/MS gliomasphere numbers (Fig. 7b), phenocopying the effects of Par3 silencing (Fig. 3a and Supplementary Fig. S2d). We then examined the role of artificial induction of ROS on gliomasphere formation (Fig. 7c, d). Treatment of U3031MG/MS cells with MitoPQ increased intracellular ROS production significantly (Fig. 7c), and caused a notable reduction in gliomasphere formation (Fig. 7d), comparable to the effect of Par3 silencing (Fig. 3a and Supplementary Fig. S2d).

The data suggest a link between Par3 and mitochondrial ROS generation, governing both positive regulation of gliomasphere formation and negative regulation of GBM cell invasiveness by this protein. We, therefore, attempted to identify whether a pool of Par3 co-localized with mitochondria in GBM cells. We employed proximity-ligation assays (PLA) as a sensitive and quantitative method to detect endogenous Par3 protein, coupled to mitochondrial decoration using MitoTracker Deep Red and fluorescence microscopy, followed by digital deconvolution and correlation analysis (Fig. 7e). PLA adapted to the detection of a single protein (instead of monitoring the interaction between two different proteins) is a well-established technique for both intracellular and extracellular proteins and has even been adopted as a clinically useful diagnostic assay for proteins of very low abundance or even extracellular vesicles in biological liquids [33–36]. Par3 puncta were readily observed in U3031MG/MS cells and were exclusively cytoplasmic, as expected (Fig. 7e). A proportion of the Par3 population was arranged in the close vicinity of the mitochondrial network in every GBM cell examined (Fig. 7e; high magnification insets with enhanced contrast). A small number of yellow spots indicating the possibility for co-localization of Par3 and mitochondria could be observed (Fig. 7e, arrows). Correlation analysis performed on 18 independent images of U3031MG/MG cells generated a Spearman coefficient of 0.67 and a Pearson coefficient of 0.48, both statistical methods supporting a relative degree of proximity between Par3 PLA signals and mitochondrial MitoTracker Deep Red signals. These data do not support a strong accumulation of Par3 protein on the cytoplasmic surface of mitochondria but are congruent with the existence of a small pool of Par3 that may interact with mitochondrial proteins.

### Enrichment of Par3 in stem-like cell populations of GBM tumor tissue

**Fig. 8:**
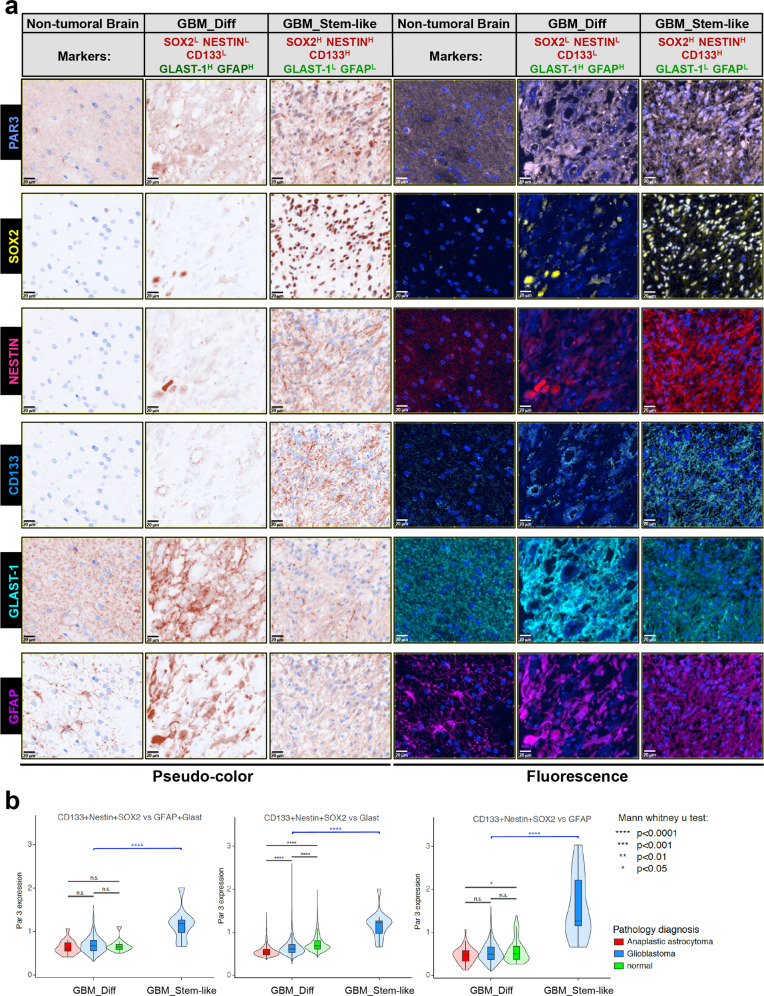
Enriched Par3-positive cells in GBM tumor tissue with stem-like cell populations. **a** Representative images displaying staining of Par3, NESTIN, CD133, SOX2, GFAP, and GLAST-1, in normal brain and GBM samples. Brown pseudo-color is shown on the left and original fluorescence color to the right. Magnification bars, 100 µm. **b** Par3 expression in normal, GBM, and anaplastic astrocytoma samples (color-coded) plotted in relation to the five marker proteins in the same tissue. Tumor cells were divided in two groups, GBM_Diff (SOX2^low^/NESTIN^low^/CD133^low^/GLAST-1^high^/GFAP^high^), shown in the figure as SOX2^L^/NESTIN^L^/CD133^L^/GLAST-1^H^/GFAP^H^ and GBM_Stem-Like (SOX2^high^/NESTIN^high^/CD133^high^/GLAST-1^low^/GFAP^low^) shown in the figure as SOX2^H^/NESTIN^H^/CD133^H^/GLAST-1^L^/GFAP^L^. Significant differences, *p < 0.05; ****p < 0.0001; n.s. not-significant.

To analyze Par3 protein expression in GBM patient stem-like cell subpopulations in situ, we performed quantitative multiplex immunohistochemical analysis using a tissue microarray (TMA) of human GBM, anaplastic astrocytoma, and non-tumoral brain samples (Fig. 8). We used antibodies against Par3, NESTIN, CD133, and SOX2, which are established stem-cell markers in GBM, as well as GFAP and glutamate aspartate transporter (GLAST)-1, as indicators of the astrocytic lineage. We then classified cells in each tumor section into two groups: GBM_diff (SOX2^low^/NESTIN^low^/CD133^low^/GLAST-1^high^/GFAP^high^) representing differentiated astrocytes, and GBM_Stem-like (SOX2^high^/NESTIN^high^/CD133^high^/GLAST-1^low^/GFAP^low^) representing rare stem-like cells. Immunostaining of non-tumoral brain tissue showed that Par3, GLAST-1, and GFAP were expressed at a moderate level, whereas the stem-cell markers NESTIN, CD133, and SOX2 were almost undetectable (Fig. 8a; note the characteristic astrocytic morphology evident after GFAP and less after GLAST-1 staining in non-tumoral brain). Interestingly, in the GBM samples, Par3 expression level was higher in tumor cells classified as GBM_Stem-like (Fig. 8). GBM cells with high GFAP and GLAST-1 (GBM_Diff group) exhibited the lowest Par3 levels (Fig. 8b). This analysis suggests that, although Par3 is expressed in normal glial cells, it is selectively enriched in cell populations that can be defined as stem-like cells in brain tumor tissue.

## Discussion

This study provides evidence for bifunctional cell biological actions of Par3 in GBM (Supplementary Fig. S6). Accordingly, the presence of Par3 in GBM cells provides a relative barrier to invasiveness (Fig. 3). Simultaneously, analyses of both in-culture cells and patient tissue support a positive role of Par3 on stem-like features of GBM (Figs. 2 and 8). A cell biological process that may explain these roles of Par3 in GBM is the regulation of mitochondrial metabolism and the homeostatic generation of ATP by the tumor cells (Figs. 4–7). The fact that glioblastoma continues to carry a poor prognosis despite current treatments, and since progressive tumor invasiveness and intracranial metastasis remain a challenge [1, 2, 4], makes this study relevant to the above areas of GBM biology.

Our focus on Par3 stems from the fact that advanced and invasive tumors are characterized by loss of cell polarity [9, 10]. Thus, since Par3 regulates polarity [12, 13], it is logical that several studies demonstrate alterations in the expression of Par3 in a range of human cancers [19, 37–39]. In agreement with this cancer-wide consensus, we report that low *PARD3* mRNA levels among GBM patient samples in public databases, correlate with poor survival of the patients (Fig. 1). Transcriptomic studies in various tumors, including GBM, also suggested potential tumor suppressor functions for Par3 [10, 19]. Our genomic analysis of seven patient-derived GBM cultures revealed SNPs but no loss-of-function mutations and decreased copy numbers of the *PARD3* locus, the latter impacting on the loss of additional important genes in the *PARD3* vicinity (Supplementary Fig. S1).

Several reports of normal neural development support our findings in GBM. For example, during neurogenesis, Par3 regulates asymmetric cell divisions and inhibits differentiation of cortical and radial glial progenitor cells [14, 15, 40]. These observations show that an enrichment of Par3 protein in GBM cells that co-express SOX2, CD133, and NESTIN (Fig. 8; GBM_Stem-like) may have biological relevance. We believe that this and equivalent studies will gradually develop the framework for the identification of GBM stem-like cells in human tumors in situ, thus assisting disease diagnosis. This is necessary as the GBM stem-like cells are difficult to identify because of their plasticity, which leads to stem-like populations shifting to non-stem-like cells and vice versa, a process also affected by drug or radiation treatments [41]. Our efforts to validate this model experimentally by silencing Par3 in patient-derived GBM cultures, do support a significant decrease in gliomasphere formation, an in vitro surrogate assay of self-renewal potential (Fig. 2 and Supplementary Fig. S2d). Further supportive to these results is the concomitant reduction in expression of GBM stemness markers (*SOX2*, *NESTIN*; Fig. 2c) caused by Par3 silencing, and the reciprocal increase in expression of astrocytic differentiation marker (*GFAP*) expression, but not neuronal marker (*βIIITub*, *MBP*), when GBM cells were cultured under physiological conditions that promote astrocyte differentiation (Fig. 2d). However, we interpret these results with certain caution, as not all stemness markers were equally affected by Par3 silencing. Whether Par3 regulates asymmetric cell divisions in GBM, as proven for developing normal glial progenitors [14, 15, 40], remains to be examined.

Although Par3 does not directly regulate gene expression, the transcriptomic analysis revealed a significant number of differentially expressed genes after Par3 silencing (Fig. 4). This finding may reflect an adaptation of GBM cells to the loss of Par3 over a period of a few days. Unexpectedly though, this analysis identified a clear functional group of genes representing enzymes responsible for ATP synthesis and family members of the mitochondrial membrane carriers/transporters, which were downregulated after Par3 silencing (Fig. 4d). Polarization is energy demanding and requires coordination with cellular energy homeostasis. In hepatocytes, cell polarization leads to increased oxidative phosphorylation, which helps raise intracellular ATP levels [42]. As the cells polarize, the mitochondrial membrane potential increases [42]. As predicted by this independent study, lowering Par3 expression in GBM cells resulted in low ATP levels (Fig. 5). This finding bridges the function of Par3 with a relatively well-established field of GBM metabolism, which has been shown to depend on mitochondrial action, glucose oxidation, and glycolysis at least in mice bearing GBM tumors [43], and in various studies of human GBM [44]. Furthermore, human GBMs with stem-like characteristics appear to generate their ATP via mitochondrial oxidative phosphorylation in parallel to the generation of ROS [45].

Mitochondria are also primary organelles for the production of ROS in diverse types of cancer. ROS activate pro-tumorigenic signaling, cell survival, autophagy and proliferation, genetic instability, and aggressive phenotype [46–48]. In our study, silencing Par3 increased the intracellular (mainly mitochondrial) levels of ROS (Fig. 5), and chemically-induced synthesis of mitochondrial ROS by MitoPQ enhanced invasiveness, thus phenocopying Par3 silencing (Figs. 6d, e and 7c, d). Such invasiveness was even blocked by treatment with anti-oxidants (Fig. 6). This result is interesting to compare to GBM cell studies where the cells responded to serum treatment by differentiating, and downregulating specific stem-like genes via ROS accumulation, whereas anti-oxidants such as NAC, preserved stem-like features [49]. In GBM, ROS may also play bifunctional roles [50]. For example, mitochondrial ROS can activate the p38 mitogen-activated protein kinase, which causes degradation of the pro-apoptotic factor BMI1 and stabilization of the transcription factor FOXO3 [51]. The last two molecular changes in response to ROS favor differentiation-like changes in GBM cells but not stem-like growth [51]. Essentially the same mechanism has been proposed by studies where treatment of GBM with cannabidiol has been analyzed [52]. The benefit that cannabidiol provided to mice bearing GBMs was based on a p38- and ROS-dependent suppression of stemness factors [52]. Characteristically, experimental GBMs exhibited resistance to this drug, by inducing an anti-oxidant response and thus preserving their stem-like features [52]. Thus, ROS generation in GBM links to new possibilities of patient treatment.

Beyond mitochondria, NADPH oxidases can generate ROS in cancer cells, enhancing invasiveness and metastasis as for example in melanoma and colorectal cancer [30, 53]. Our study did not examine the link between Par3 and the functions of NADPH oxidases. It is also notable that the increase in intracellular ROS caused by Par3 silencing did not cause apoptosis or autophagy (Supplementary Fig. S3). These observations may indicate a more direct mechanism of mitochondrial ROS regulation by Par3 function in GBM. However, our observations using an aPKC inhibitor and in situ PLA analysis (Figs. 6f and 7) suggest the need for a screen to identify partners of Par3 that physically link the Par3/aPKC activity with mitochondrial enzymatic functions. Since Par3 localization at the mitochondrial membrane remains possible (Fig. 7), we favor the possibility of intermediate mediators.

In conclusion, our study demonstrates that, on one hand, Par3 acts in an oncogenic manner by supporting gliomasphere formation by GBM cells (Supplementary Fig. S6). On the other hand, Par3 prevents invasiveness of the same GBM cells into the surrounding extracellular matrix (Supplementary Fig. S6). We have thus established a dual functional role of the polarity protein Par3, in glioblastoma.

## Materials and methods

All commercial suppliers are cited fully only the first time that they are cited.

### GBM cell culture and treatments

The patient-derived GBM cultures were obtained from the HGCC resource of Uppsala University, Sweden, which authenticates all primary cultures [25]. U3005/3013/3024/3028/3031/3034/3062MG passages 15−30 were cultured in N2B27 media (Thermo Fischer Scientific, Uppsala, Sweden) [Dulbecco’s Modified Eagle’s Medium (DMEM)/F12 Glutamax and Neurobasal medium [53] mixed at 1:1 ratio, with the addition of 1% B27 and 1% N2, 100 U/ml penicillin and 100 mg/ml streptomycin (Sigma-Aldrich Sweden AB, Stockholm, Sweden), 10 ng/ml EGF and 10 ng/ml FGF2 (PeproTech, EC Ltd, London, UK)]. Adherent cultures were seeded onto poly-ornithine/laminin-coated dishes and passaged as described [54]. Cells were treated with 1 µM aPKCi, (CRT-0066854-hydrochloride; TOCRIS/Bio-Techne Ltd, Abingdon, UK), 100 µM MitoPQ (mitochondria-targeted redox cycler; Abcam, Cambridge, UK) or their vehicle, dimethyl-sulfoxide (DMSO), and with 50 µM MitoTEMPO (SML0737; Sigma-Aldrich) or its vehicle, H_2_O, as indicated below and in the figures

### *PARD3* sequence analysis

Genomic DNA was isolated from each GBM line using the DNeasy (blood and tissue) kit (Qiagen AB, Sollentuna, Sweden), and was subjected to amplification on a CFX96 real time system (Bio-Rad Laboratories AB, Solna, Sweden) using the HiFi Hot Start PCR Kit (Kapa Biosystems, Roche, Solna, Sweden), with the following conditions: 95 °C for 5 min, 35 × [98 °C for 20 s, 60 °C for 30 s, 72 °C 30 s (≤300 bp amplicon)/60 s (300−900 bp amplicon)/90 s (900−1,200 bp amplicon)], 72 °C for 10 min with specific primers (Supplementary Table I). The expected size of each amplicon was verified by 1.5% w/v agarose gel electrophoresis (Supplementary Table I). PCR products from each DNA sample were pooled in equimolar quantities. Sequencing libraries for ION Proton sequencing (Thermo Fischer Scientific) were created and barcoded for de-multiplexing of samples after joint sequencing. DNA sequences were aligned and analyzed for SNP location, allelic frequency, and impact on coding capacity (synonymous or non-synonymous SNPs) using the University of California Santa Cruz Genome Browser (https://genome.ucsc.edu/). In all analyses, the human genome (hg38) *PARD3* mRNA variant 2 (NM_001184785, 5,971 nt, Supplementary Fig. S1a) and corresponding Par3 protein (NP_001171714, 1,363 amino acids, Supplementary Fig. S1b) were used as reference.

### Transient transfections

Cells were transiently transfected when 80% confluent using siRNAs (20 nM each (Supplementary Table II); Dharmacon/GE Healthcare, Uppsala, Sweden) against a non-specific target (siControl) or Par3, using Silentfect (Bio-Rad Laboratories). Twenty-four hours after transfection, dissociated cells were seeded for experiments in N2B27 medium for 2−6 days prior to functional assays.

### Extreme limiting dilution assay

Single transfected cell suspensions with siControl or siPar3 were seeded on low attachment 96-well plates in decreasing serial dilutions (64-1 cells/well), in seven replicates per condition and gliomaspheres were analyzed using the online ELDA analysis program (http://bioinf.wehi.edu.au/software/elda) [55]. For chemical interventions, cells were incubated in the presence of 1 µM aPKCi, 100 µM MitoPQ, 50 µM MitoTEMPO or vehicles (DMSO or H_2_O), for the period of sphere formation.

### Cell viability and proliferation assays

The proliferation of 5,000 cells/well seeded in a 96-well plate and transfected with control and Par3 siRNAs was monitored at 1, 3, and 6 days by MTS assay, following the manufacturer’s protocol (Promega, Biotech AB, Nacka, Sweden). For quantification of Ki67-positive cells, 15,000 cells were seeded in 8-well chambered slides for 1 or 3 days, fixed with 4% paraformaldehyde in phosphate-buffered saline (PBS) pH 7.2 for 30 min at room temperature, and incubated with Ki67 antibody (Supplementary Table III) over-night at 4 °C; after washes with PBS, cells were incubated with secondary antibody (Supplementary Table III) for 1 h at room temperature. Nuclei were stained with 2-(4-amidinophenyl)-1H-indole-6-carboxamidine (DAPI; Sigma-Aldrich) and cells were mounted with Fluoromount-G (Southern Biotech, AH diagnostics, Solna, Sweden). Ten random pictures taken with a Nikon Eclipse 90i microscope at the same exposure were quantified by ImageJ64 10.2 software (National Institutes of Health, Bethesda, MD, USA) and a quantification script (Supplementary Methods).

### Fluorimetry

For caspase-3 activity, mitochondrial assays, and ROS analysis, fluorescence was measured in a Microplate Reader Enspire (PerkinElmer Sverige AB, Upplands Väsby, Sweden), and was normalized to the corresponding protein amount in the respective cell lysate. Protein concentration was determined using Bradford Reagent (Bio-Rad Laboratories). Auto-fluorescence generated by Hank’s balanced salt solution (HBSS) without phenol red was subtracted. All fluorimetric results are expressed as fluorescence units per µg protein and then expressed as a percentage of control, unless indicated otherwise.

### Analysis of caspase-3 activity

Transfected cells with control and Par3 siRNAs were lysed in 0.5% Triton X-100, 5 mM Tris-HCl, pH 8.0, 20 mM EDTA, for caspase-3 activity, a reaction containing 20−40 μg protein extract and 20 μM fluorogenic caspase-3 substrate (Ac-DEVD-AMC, excitation at 380 nm, emission at 440 nm; BD Biosciences, Stockholm, Sweden) in 40 mM HEPES, pH 7.5, 20% glycerol, 4 mM dithiothreitol buffer, was incubated for 2 h in the dark at 37 °C, and analyzed fluorimetrically. A unit of caspase-3 activity is the amount of active enzyme causing an increase in 1 fluorescence unit and results are presented as arbitrary units of caspase-3 activity/h/μg protein.

### Mitochondrial transmembrane potential analysis

MitoTracker Red CMXROS (500 nm; excitation at 579 nm, emission at 599 nm; Thermo Fischer Scientific) was loaded into the cells by incubation in HBSS without phenol red for 30 min at 37 °C. Detached cells were resuspended in HBSS containing 500 nM MitoTracker Red CMXROS and analyzed in duplicate via fluorimetry.

### Intracellular redox state analysis

Transfected cells with control and Par3 siRNAs or incubated with DMSO, 1 µM aPKCi or 100 µM MitoPQ for 1 or 3 days, were incubated with 2.5 µM DCFH-DA (excitation at 495 nm, emission at 520 nm; Life Technologies Europe BV) in HBSS without phenol red for 30 min at 37 °C. Intracellular esterases convert DCFH-DA to 2′,7′-dichlorodihydrofluorescein that in turn is converted into 2′,7′-dichlorofluorescein when oxidized by H_2_O_2_. Cells were lysed in a 25 mM Hepes pH 7.5, 60 mM NaCl, 1.5 mM MgCl_2_, 0.2 mM EDTA, 1% Triton-X-100 solution for 10 min at 4 °C followed by fluorimetry.

### Mitochondrial ROS measurements

The MitoSOX-Red mitochondrial superoxide indicator (excitation at 510 nm, emission at 580 nm; Life Technologies) measured mitochondrial superoxide, generated as a byproduct of oxidative phosphorylation. Cells loaded with 5 µM MitoSOX-Red by incubation in HBSS without phenol red for 10 min at 37 °C, were lysed in 25 mM Hepes pH 7.5, 60 mM NaCl, 1.5 mM MgCl_2_, 0.2 mM EDTA, 1% Triton-X-100 for 10 min at 4 °C followed by fluorimetry.

### Extracellular ROS measurement

Transfected cells (75,000/well) were seeded and extracellular H_2_O_2_ was measured after 3 days directly on intact cells, using Amplex Ultra Red (excitation, 530 nm, emission, 590 nm; ThermoFisher Scientific) as an electron donor for horseradish peroxidase reactions. Amplex Ultra Red (50 µM) and horseradish peroxidase (0.1 U/ml) in HBSS without phenol red were added to the cellular samples for 2 h followed by fluorimetry in 100 µl of conditioned medium.

### Intracellular ATP analysis

Transfected cells (150,000/well) were scraped, centrifuged at 2,500 rpm at 4 °C, lysed in H_2_O, and boiled for 5 min. The ATP Determination Kit (Thermo Fischer Scientific), based on the manufacturer’s protocol, measured ATP level which is expressed as ATP per µg protein and as a percentage of control.

### Autophagy measurement with Cyto-ID

Autophagic vacuoles were measured using the Cyto-ID detection kit (Enzo Life Sciences, Solna, Sweden) in cells transfected with control or Par3 siRNAs, and treated with 40 µM chloroquine for the last 16 h of the experiments. Three days after transfection, harvested cells washed and stained for 30 min with Cyto-ID dye, were analyzed using a BD Accuri CG Plus flow cytometer (BD Biosciences) and quantified by FlowJo software version 10.4.2.

### Transwell invasion assay

Transwell plate inserts (6.5 mm diameter, 8 µm pore; Corning Costar, NY, USA), were coated with 10 μg/ml laminin (Sigma-Aldrich) for 30 min at 37 °C. Untransfected or transfected cells (5 × 10^4^) were seeded in the upper chamber in serum-free DMEM, and DMEM/6% FBS was placed in the lower chamber. Untransfected cells in the upper chamber were treated with DMSO, 1 µM aPKCi or 100 µM MitoPQ, and siRNA-transfected cells were treated with 50 µM MitoTEMPO or H_2_O. After 15 h, cells in the upper chamber were removed by a cotton swab, thereafter cells migrated through the filter were fixed with ice-cold methanol, their nuclei stained with DAPI (Sigma-Aldrich), counted using ImageJ in 10−15 pictures of each insert, and expressed as cells per invasion field.

### Collagen invasion assay

Gliomaspheres were formed using the hanging drop method [56], collected, and resuspended in a collagen I solution (1.7 mg/ml) in minimum essential medium (MEM), that polymerized at 37 °C. MEM without or with 3% FBS was added on top of the collagen, and DIC microscopic pictures of embedded spheres were taken at 0 and 48 h of incubation. For quantification of invasive growth, the increase in the area occupied by the collagen-invading cells was calculated by subtracting the spheroid core from the total area covered by cells, using ImageJ.

### Zebrafish invasion assay

Fish staging, embryo production, and preparation were conducted as described [57]. Transfected cells were stained with 4 ng/µl CM-Dil Dye (ThermoFisher Scientific) for 4 min at 37 °C, followed by 15 min at 4 °C, then cells were centrifuged for 5 min at 1,200 rpm and re-suspended in media, centrifuged again and washed twice with PBS, before single cells were suspended in PBS and stored at 4 °C prior to implantation. Cell suspensions loaded into borosilicate glass capillary needles (1 mm O.D. × 0.78 mm, I.D, Harvard Apparatus, Holliston, MA, USA) were injected in the duct of Cuvier (400 cells/embryo) of Tg(*Fli1*:EGFP) zebrafish embryos mounted on a 10-cm Petri dish coated with 1% agarose, using a Pneumatic Picopump and a manipulator (WPI, Stevenage, UK). Injected embryos were maintained at 33 °C for 6 days, followed by fixation in 4% paraformaldehyde for 2 h at room temperature, and imaging in PBS with 0.1% Tween-20 (Merck, Amsterdam, Netherlands). Fluorescent images were acquired with a Leica SP5 STED confocal microscope (Leica, Rijswijk, Netherlands). The fish population sample size was determined based on the power for discrimination between conditions and was estimated empirically based on preliminary results. Based on the observed differences, 21 fish per condition were sufficient. Fish were randomly assigned for injection experiments with different populations of cancer cells. Blinding of the fish experiments was not possible. Every individual animal was followed during the course of tumor cell implantation and circulation with equal attention and determination.

### RNA extraction and expression analysis

RNA from ~250,000 cells was purified and gene expression was analyzed by real-time RT-PCR in a CFX Connect instrument (Bio-Rad Laboratories AB, Solna, Sweden) as described [58], with specific primers (Supplementary Table IV).

RNA-Seq analysis was performed on an Ion Proton System for next-generation sequencing (Life Technologies), with triplicate samples (10 ng RNA) per condition. The sequence reads were analyzed using the AmpliSeqRNA analysis plugin, v4.2.1, in the Torrent Suite Software (Life Technologies), counting the number of sequences obtained for all cDNA amplicons. The resulting counts represent gene expression levels for 20,800 different genes present in the AmpliSeq Human Gene Expression panel. Expression level counts for all samples were merged into a table, used for differential gene expression (DE) analysis with the R/Bioconductor package EdgeR (http://www.bioconductor.org/ [59]), using standard parameters. Adjusted *p*-values (padj) for multiple testing were calculated for final estimation of DE significance, using Benjamini-Hochberg to estimate the false discovery rate (FDR), followed by functional enrichment using the R package clusterProfiler (http://www.bioconductor.org/ [60]) and Enrichr [29]. Primary data, deposited to Array Express (accession number E-MTAB-7724) are presented in Supplementary Table V (and sub-tables).

### Immunoblot analysis of protein expression

Total proteins from ~600,000 cells were extracted and analyzed by immunoblotting using the antibodies specified in Supplementary Table III as described [58]. Densitometric quantification was performed using ImageJ. Protein band density was measured as a differential from the surrounding “empty” area of the immunoblot, normalized against the corresponding loading control (GAPDH/β-actin), and expressed as 1 under the basal or control condition, except in Fig. 1c, where relative protein intensities are presented without normalization.

### Super-resolution confocal microscopy and proximity-ligation assay

Transfected cells were incubated in 200 nM MitoTracker Deep Red (Thermo Fischer Scientific) according to the manufacturer’s protocol. Stained cells, mounted in ProLong Glass Antifade Mountant (Thermo Fischer Scientific) were analyzed in a structured illumination microscopy (SIM) super-resolution microscope Zeiss ELYRA-S.1 (Carl Zeiss AB, Stockholm, Sweden). Mitochondrial branch length and footprints were analyzed using ImageJ plug-in MiNA [61].

PLA was performed on cells stained with MitoTracker Deep Red (Thermo Fischer Scientific) using Duolink PLA products (Sigma-Aldrich) according to a standard protocol at the PLA and Single Cell Proteomics Facility SciLifeLab. Briefly, cells fixed with 3.7% paraformaldehyde were blocked at 37 °C for 1 h in Duolink Blocking Solution, incubated at 4 °C overnight with anti-Par3 antibody (Supplementary Table III) diluted in Duolink Antibody Diluent, washed 3 times for 5 min in TBS/0.05% Tween-20 (TBST), followed by 1 h incubation with Duolink PLA secondary probes anti-rabbit PLUS (DUO92002) and anti-rabbit MINUS (DUO92005) at 37 °C. The cells were washed 3 × 5 min in TBST and incubated for 30 min at 37 °C in ligation/hybridization solution, followed by 3 × 5 min washes in TBST. The PLA signal was amplified by rolling-circle amplification using phi29 Polymerase in amplification solution (solutions prepared as described [62]). Detection oligonucleotides were labeled with Texas Red and nuclei with DAPI. After water rinsing, slides were mounted with SlowFade Gold Antifade Reagent (Thermo Fisher Scientific). Negative controls were incubated with rabbit IgGs instead of primary antibody (Sigma-Aldrich). Images were acquired with a Zeiss Axio Imager Z2 microscope and Huygens Essential software (SVI, Netherlands) was used for image deconvolution and co-localization analysis.

### Multiplex immunohistochemical staining

A TMA that contained 35 samples of independent glioblastoma and astrocytoma patients and five samples of normal brain tissue, each in duplicate, generating 80 tissue cores (TMA-GL806d; US Biomax, Derwood, MF, USA), was analyzed by multiplexed immunohistochemistry as described [63] with adaptations. For antigen retrieval, slides were boiled in pH 9.0 buffer (AR6001, PerkinElmer) for 15 min, using a microwave oven, and primary antibodies are listed in Supplementary Table III. Incubation with anti-rabbit/mouse Opal Polymer HRP ready-to-use immunohistochemistry detection reagent (ARH1001EA, PerkinElmer) for 10 min and Opal fluorophores (Supplementary Table III) for 10 min at room temperature followed. DAPI staining and mounting with ProlongTM Diamond Antifade Mountant (Thermo Fisher Scientific) completed the protocol.

Using the Vectra Polaris (PerkinElmer) multispectral imaging mode, the TMA cores were scanned, and images were analyzed by inForm software by applying spectral unmixing, cell segmentation and recording mean expression levels of each antigen in every cell. Imaging data analysis was performed by the version 3.3.3 of the R software [64, 65]. Protein expression ranged in three levels; ‘low’ level was considered if the antibody intensity was below a threshold, visually defined as background. Then, the remaining intensity range (from visually defined background to maximal) was split into two groups, using the mean level as cut-off. The expression above the background but below the mean cut-off was considered as ‘medium’, and intensity above the mean cut-off as ‘high’. These data were used to define two classes with regard to cell differentiation status in the tumors: GBM_Diff (SOX2^low^/NESTIN^low^/CD133^low^/ GLAST-1^high^/GFAP^high^) representing differentiated astrocytes in normal brain tissue, and GBM_Stem-Like (SOX2^high^/NESTIN^high^/CD133^high^/GLAST-1^low^/GFAP^low^) representing stem-like tumor cells. Par3 intensity was normalized by setting the background threshold to ‘zero’ intensity. Cells with Par3 signal below background threshold were excluded from analysis, resulting in 55,952 cells used for analysis. Statistical difference between Par3 levels in different cell subgroups was calculated by Mann−Whitney U-rank test.

### Statistical analysis and repeatability of experiments

Data were analyzed using Prism GraphPad v6.0. A two-tailed Student *t*-test was performed in two-group comparisons where data exhibited similar variance between compared groups. The data also met the assumption of the normal distribution. Additional statistical methods are described in the method sections. In the figures, when SEM is reported, it has been calculated based on the independent experiments (*n* = number of experiments) as reported in the figure legends.

## Supplementary information

Supplementary File

Supplementary Table V

## Data Availability

The Ampliseq RNA sequencing primary data are deposited to Array Express, EBI, UK under accession number E-MTAB-7724, and are also presented in Supplementary Table V (and sub-tables). A simple quantification code presented used for image analysis is presented in its entirety in the Supplementary Methods. All additional primary data generated or analyzed during this study are included in this published article (and its supplementary information files) or are available from the corresponding author on reasonable request.

## References

[1] Davis FG, Smith TR, Gittleman HR, Ostrom QT, Kruchko C, Barnholtz-Sloan JS (2020). Glioblastoma incidence rate trends in Canada and the United States compared with England, 1995-2015. Neuro Oncol.

[2] Ostrom QT, Cioffi G, Gittleman H, Patil N, Waite K, Kruchko C (2019). CBTRUS statistical report: primary brain and other central nervous system tumors diagnosed in the United States in 2012−2016. Neuro Oncol.

[3] Stupp R, Hegi ME, Mason WP, van den Bent MJ, Taphoorn MJ, Janzer RC (2009). Effects of radiotherapy with concomitant and adjuvant temozolomide versus radiotherapy alone on survival in glioblastoma in a randomised phase III study: 5-year analysis of the EORTC-NCIC trial. Lancet Oncol.

[4] Westermark B (2012). Glioblastoma-a moving target. Ups J Med Sci.

[5] Wang Q, Hu B, Hu X, Kim H, Squatrito M, Scarpace L (2017). Tumor evolution of glioma-intrinsic gene expression subtypes associates with immunological changes in the microenvironment. Cancer Cell.

[6] Verhaak RG, Hoadley KA, Purdom E, Wang V, Qi Y, Wilkerson MD (2010). Integrated genomic analysis identifies clinically relevant subtypes of glioblastoma characterized by abnormalities in PDGFRA, IDH1, EGFR, and NF1. Cancer Cell.

[7] Gimple RC, Bhargava S, Dixit D, Rich JN (2019). Glioblastoma stem cells: lessons from the tumor hierarchy in a lethal cancer. Genes Dev.

[8] Singh SK, Hawkins C, Clarke ID, Squire JA, Bayani J, Hide T (2004). Identification of human brain tumour initiating cells. Nature.

[9] Macara IG, McCaffrey L (2013). Cell polarity in morphogenesis and metastasis. Philos Trans R Soc Lond B Biol Sci.

[10] Fomicheva M, Tross EM, Macara IG (2020). Polarity proteins in oncogenesis. Curr Opin Cell Biol.

[11] St Johnston D (2018). Establishing and transducing cell polarity: common themes and variations. Curr Opin Cell Biol.

[12] Vorhagen S, Niessen CM (2014). Mammalian aPKC/Par polarity complex mediated regulation of epithelial division orientation and cell fate. Exp Cell Res.

[13] Hapak SM, Rothlin CV, Ghosh S (2018). PAR3-PAR6-atypical PKC polarity complex proteins in neuronal polarization. Cell Mol Life Sci.

[14] Bultje RS, Castaneda-Castellanos DR, Jan LY, Jan YN, Kriegstein AR, Shi SH (2009). Mammalian Par3 regulates progenitor cell asymmetric division via notch signaling in the developing neocortex. Neuron.

[15] Sottocornola R, Royer C, Vives V, Tordella L, Zhong S, Wang Y (2010). ASPP2 binds Par-3 and controls the polarity and proliferation of neural progenitors during CNS development. Dev Cell.

[16] Lyu J, Kim HR, Yamamoto V, Choi SH, Wei Z, Joo CK (2013). Protein phosphatase 4 and Smek complex negatively regulate Par3 and promote neuronal differentiation of neural stem/progenitor cells. Cell Rep.

[17] Chen S, Chen J, Shi H, Wei M, Castaneda-Castellanos DR, Bultje RS (2013). Regulation of microtubule stability and organization by mammalian Par3 in specifying neuronal polarity. Dev Cell.

[18] Xu G, Wang R, Wang Z, Lei Q, Yu Z, Liu C (2015). NGL-2 is a new partner of PAR complex in axon differentiation. J Neurosci.

[19] Lin WH, Asmann YW, Anastasiadis PZ (2015). Expression of polarity genes in human cancer. Cancer Inf.

[20] Rothenberg SM, Mohapatra G, Rivera MN, Winokur D, Greninger P, Nitta M (2010). A genome-wide screen for microdeletions reveals disruption of polarity complex genes in diverse human cancers. Cancer Res.

[21] Cancer Genome Atlas Research N. (2008). Comprehensive genomic characterization defines human glioblastoma genes and core pathways. Nature.

[22] Gravendeel LA, Kouwenhoven MC, Gevaert O, de Rooi JJ, Stubbs AP, Duijm JE (2009). Intrinsic gene expression profiles of gliomas are a better predictor of survival than histology. Cancer Res.

[23] Madhavan S, Zenklusen JC, Kotliarov Y, Sahni H, Fine HA, Buetow K (2009). Rembrandt: helping personalized medicine become a reality through integrative translational research. Mol Cancer Res.

[24] Bowman RL, Wang Q, Carro A, Verhaak RG, Squatrito M (2017). GlioVis data portal for visualization and analysis of brain tumor expression datasets. Neuro Oncol.

[25] Xie Y, Bergström T, Jiang Y, Johansson P, Marinescu VD, Lindberg N (2015). The human glioblastoma cell culture resource: validated cell models representing all molecular subtypes. EBioMedicine.

[26] Lottaz C, Beier D, Meyer K, Kumar P, Hermann A, Schwarz J (2010). Transcriptional profiles of CD133+ and CD133- glioblastoma-derived cancer stem cell lines suggest different cells of origin. Cancer Res.

[27] Vitale I, Manic G, Dandrea V, De Maria R (2015). Role of autophagy in the maintenance and function of cancer stem cells. Int J Dev Biol.

[28] Mescher M, Jeong P, Knapp SK, Rubsam M, Saynisch M, Kranen M (2017). The epidermal polarity protein Par3 is a non-cell autonomous suppressor of malignant melanoma. J Exp Med.

[29] Kuleshov MV, Jones MR, Rouillard AD, Fernandez NF, Duan Q, Wang Z (2016). Enrichr: a comprehensive gene set enrichment analysis web server 2016 update. Nucleic Acids Res.

[30] Moloney JN, Cotter TG (2018). ROS signalling in the biology of cancer. Semin Cell Dev Biol.

[31] Sabharwal SS, Schumacker PT (2014). Mitochondrial ROS in cancer: initiators, amplifiers, or an Achilles’ heel?. Nat Rev Cancer.

[32] Robb EL, Gawel JM, Aksentijevic D, Cocheme HM, Stewart TS, Shchepinova MM (2015). Selective superoxide generation within mitochondria by the targeted redox cycler MitoParaquat. Free Radic Biol Med.

[33] Björkesten J, Enroth S, Shen Q, Wik L, Hougaard DM, Cohen AS (2017). Stability of proteins in dried blood spot biobanks. Mol Cell Proteom.

[34] Darmanis S, Nong RY, Vanelid J, Siegbahn A, Ericsson O, Fredriksson S (2011). ProteinSeq: high-performance proteomic analyses by proximity ligation and next generation sequencing. PLoS One.

[35] Fredriksson S, Gullberg M, Jarvius J, Olsson C, Pietras K, Gustafsdottir SM (2002). Protein detection using proximity-dependent DNA ligation assays. Nat Biotechnol.

[36] Larssen P, Wik L, Czarnewski P, Eldh M, Lof L, Ronquist KG (2017). Tracing cellular origin of human exosomes using multiplex proximity extension assays. Mol Cell Proteom.

[37] Huang L, Muthuswamy SK (2010). Polarity protein alterations in carcinoma: a focus on emerging roles for polarity regulators. Curr Opin Genet Dev.

[38] McCaffrey LM, Montalbano J, Mihai C, Macara IG (2012). Loss of the Par3 polarity protein promotes breast tumorigenesis and metastasis. Cancer Cell.

[39] Nakamura H, Nagasaka K, Kawana K, Taguchi A, Uehara Y, Yoshida M (2016). Expression of Par3 polarity protein correlates with poor prognosis in ovarian cancer. BMC Cancer.

[40] Costa MR, Wen G, Lepier A, Schroeder T, Gotz M (2008). Par-complex proteins promote proliferative progenitor divisions in the developing mouse cerebral cortex. Development.

[41] Safa AR, Saadatzadeh MR, Cohen-Gadol AA, Pollok KE, Bijangi-Vishehsaraei K (2015). Glioblastoma stem cells (GSCs) epigenetic plasticity and interconversion between differentiated non-GSCs and GSCs. Genes Dis.

[42] Fu D, Mitra K, Sengupta P, Jarnik M, Lippincott-Schwartz J, Arias IM (2013). Coordinated elevation of mitochondrial oxidative phosphorylation and autophagy help drive hepatocyte polarization. Proc Natl Acad Sci USA.

[43] Marin-Valencia I, Yang C, Mashimo T, Cho S, Baek H, Yang XL (2012). Analysis of tumor metabolism reveals mitochondrial glucose oxidation in genetically diverse human glioblastomas in the mouse brain in vivo. Cell Metab.

[44] Iranmanesh Y, Jiang B, Favour OC, Dou Z, Wu J, Li J (2021). Mitochondria’s role in the maintenance of cancer stem cells in glioblastoma. Front Oncol.

[45] van Noorden CJF, Hira VVV, van Dijck AJ, Novak M, Breznik B, Molenaar RJ (2021). Energy metabolism in IDH1 wild-type and IDH1-mutated glioblastoma stem cells: a novel target for therapy?. Cells.

[46] Banskota S, Regmi SC, Kim JA (2015). NOX1 to NOX2 switch deactivates AMPK and induces invasive phenotype in colon cancer cells through overexpression of MMP-7. Mol Cancer.

[47] Kumar B, Koul S, Khandrika L, Meacham RB, Koul HK (2008). Oxidative stress is inherent in prostate cancer cells and is required for aggressive phenotype. Cancer Res.

[48] Wang HP, Wang X, Gong LF, Chen WJ, Hao Z, Feng SW (2016). Nox1 promotes colon cancer cell metastasis via activation of the ADAM17 pathway. Eur Rev Med Pharm Sci.

[49] Yuan S, Lu Y, Yang J, Chen G, Kim S, Feng L (2015). Metabolic activation of mitochondria in glioma stem cells promotes cancer development through a reactive oxygen species-mediated mechanism. Stem Cell Res Ther.

[50] Olivier C, Oliver L, Lalier L, Vallette FM (2020). Drug resistance in glioblastoma: the two faces of oxidative stress. Front Mol Biosci.

[51] Sato A, Okada M, Shibuya K, Watanabe E, Seino S, Narita Y (2014). Pivotal role for ROS activation of p38 MAPK in the control of differentiation and tumor-initiating capacity of glioma-initiating cells. Stem Cell Res Ther.

[52] Singer E, Judkins J, Salomonis N, Matlaf L, Soteropoulos P, McAllister S (2015). Reactive oxygen species-mediated therapeutic response and resistance in glioblastoma. Cell Death Dis.

[53] Aydin E, Johansson J, Nazir FH, Hellstrand K, Martner A (2017). Role of NOX2-derived reactive oxygen species in NK cell-mediated control of murine melanoma metastasis. Cancer Immunol Res.

[54] Pollard SM, Yoshikawa K, Clarke ID, Danovi D, Stricker S, Russell R (2009). Glioma stem cell lines expanded in adherent culture have tumor-specific phenotypes and are suitable for chemical and genetic screens. Cell Stem Cell.

[55] Hu Y, Smyth GK (2009). ELDA: extreme limiting dilution analysis for comparing depleted and enriched populations in stem cell and other assays. J Immunol Methods.

[56] Ware MJ, Colbert K, Keshishian V, Ho J, Corr SJ, Curley SA (2016). Generation of homogenous three-dimensional pancreatic cancer cell spheroids using an improved hanging drop technique. Tissue Eng Part C Methods.

[57] Ren J, Liu S, Cui C, Ten Dijke P. Invasive behavior of human breast cancer cells in embryonic zebrafish. J Vis Exp. 2017. 10.3791/55459. 10.3791/55459 PMC5565102 28518096

[58] Caja L, Tzavlaki K, Dadras MS, Tan E-J, Hatem G, Maturi NP (2018). Snail regulates BMP and TGFβ pathways to control the differentiation status of glioma-initiating cells. Oncogene.

[59] Robinson MD, McCarthy DJ, Smyth GK (2010). edgeR: a Bioconductor package for differential expression analysis of digital gene expression data. Bioinformatics.

[60] Yu G, Wang LG, Han Y, He QY (2012). clusterProfiler: an R package for comparing biological themes among gene clusters. OMICS.

[61] Valente AJ, Maddalena LA, Robb EL, Moradi F, Stuart JA (2017). A simple ImageJ macro tool for analyzing mitochondrial network morphology in mammalian cell culture. Acta Histochem.

[62] Söderberg O, Gullberg M, Jarvius M, Ridderstrale K, Leuchowius KJ, Jarvius J (2006). Direct observation of individual endogenous protein complexes in situ by proximity ligation. Nat Methods.

[63] Mezheyeuski A, Bergsland CH, Backman M, Djureinovic D, Sjoblom T, Bruun J (2018). Multispectral imaging for quantitative and compartment-specific immune infiltrates reveals distinct immune profiles that classify lung cancer patients. J Pathol.

[64] Team R. Integrated development for R. RStudio, Inc, Boston, MA, USA. http://www.rstudio.com/. 2015.

[65] Team RC. R: A language and environment for statistical computing. R Foundation for Statistical Computing, Vienna, Austria. http://www.R-project.org/. 2017.

