# Supplementary material for: The polarity protein Par3 coordinates positively self-renewal and negatively invasiveness in glioblastoma
Source: Cell Death Dis. 2021 Oct 12;12(10):932. doi: 10.1038/s41419-021-04220-7 (PMC8511086; doi:10.1038/s41419-021-04220-7)
Supplement: Supplementary file 1 — Supplementary File [file 41419_2021_4220_MOESM1_ESM.pdf]

Par3 promotes glioblastoma stem cell self-renewal while inhibiting cell invasion

Mahsa Shahidi Dadras, Laia Caja, Sijia Liu, Artur Mezheyeuski, Caroline Gélabert, Maria Catalina Gomez-Puerto, Radiosa Gallini, Peter ten Dijke, Carl-Johan Rubin, Carl-Henrik Heldin and Aristidis Moustakas

Supplementary Information

Supplementary Methods

Quantification of Ki67-postive cells

The following script was used in ImageJ64 10.2 software in order to automate the quantification of Ki67-positive nuclei and the total number of cells.

```
//image tiff
idimage=getImageID(); //rename(Image);
getTitle();
print ("Image", idimage);
run("Stack to Images");
selectWindow("Red");
close();
selectWindow("Blue");
setAutoThreshold("Otsu dark");
setOption("BlackBackground", true);
run("Convert to Mask");
run("Watershed");
run("Set Measurements...", "area mean min limit display redirect=None decimal=1");
```

```
run("Analyze Particles...", "size=100-Infinity display exclude include summarize add");
close();
selectWindow("Green");
setAutoThreshold("Intermodes dark");
run("Convert to Mask");
run("Options...", "iterations=9 count=1 black do=Dilate");
run("Watershed");
run("Analyze Particles...", "size=100-Infinity display exclude include summarize add");
close();
```

---

end of script.

Supplementary Table I. DNA primers used to sequence the *PARD3* exome

Exon nr	Exon length (bp)	Forward primer (5´-3´)	Reverse primer (5´-3´)	Amplicon length (bp)
1	421	TTCCAGGAAGCGCCATATT	ATGTGCGCGGTGTTGTGT	606
2	102	CCGCTCCCTAGTCCTCAAA	ATGGGCAGGAAGTTATCACG	256
3	181	GAACATTTGCCTGGGTCCTA	TGTTTCTTTGAGGTTTTTCTTCG	474
4	179	ACACTCATCACGGACACCAA	CCCACTTGTCCCCTTTTACA	375
5	132	TGTTACAGCAATGACGCACA	CGAGGTCTAATCTGGGTGGA	341
6	92	CCTCATGCCATATGCTTTCA	CTTTGTGTTGGCTGCCTCTA	201
7	84	AGCAGTTGCCTCCAAATGAG	TCATGCTCCCTCATTCATCA	245
8	126	AGTGCAGCGAGCACACACT	GTAAGGCAGTGTGCATGGTG	210
9	383	CCCTTGTGTGTTGCTGCTAA	TTCAATTTCAACAGCCTGTCA	560
10	140	AATCAGGGAACATCCTGCTG	GAAGAAATGTGGACCATTGTG	299
11	129	TCAGGCAACTAACCCAGACC	TTCCCAGATCATGGCAGAAT	359
12	39	TCCAACATCTCTGCATCCTTC	TGTGTTGAAGATGGCTTTGG	134
13	189	GGCATACGCATGATAAAGTTGA	ATGGATCTTGCTGTGGAACC	355
14	171	AACACAAACCACAATCCACCT	CGTTCAATTGGGCATAATCC	486
15	159	TCAGTAAACCAATGAAAGCATCA	TGGCCTCCAGGTACTGATTT	261
16	190	ACCCTGGACGATGAGCTTAC	CAGGTTGAGCAGATATGTGGAA	330
17	152	CTGCTTGGGACCATCAATTT	GCAGGTATGTATGAACCTCCCTA	309
18	45	CTATGTGGGCCATCGTTTCT	TGGTGGGCTATGGGAACTAA	148
19	228	TACAGGTCTCCATGCCTTCA	TCTTTTCCATCTGCATTCCA	365
20	232	TGGTTTGGGATGGTAACGAC	TTGTTGCGTTGGTTCACTGT	305
21	111	CCCACAACCATCAATATTTGG	CTGCAGAAGACACCTGATCC	384
22	243	TGGAAGCAATACCACGATTG	TACCACTGATCTTCTATAGC	404
23	121	GTAGGACCATTCACCGTGCT	CAGCAAGTTGCCATGTTTTG	258
24	129	GAACAGTAGCCACGGGACAT	GAATCGAAATGGAGTCAACCA	360
25 (a)	2,002	CACTGATGCTAGGTGCTCCA	AGGAACTGGTGGTTGATTGC	521
25 (b)	2,002	GCAATCAACCACCAGTTCCT	CCCGTGTCTCTTCTGCATTT	525
25 (c)	2,002	CCGCACGTGATTAAGAAACA	CTCATGGCCCAGTACATCCT	630
25 (d)	2,002	AGGATGTACTGGGCCATGAG	ACCTGAAGTGGAGGATGACG	515
25 (e)	2,002	TGCAGGGATGTTACAACCAA	GGAAGGAGCAGCAGATGAAG	517
25 (f)	2,002	CTTCATCTGCTGCTCCTTCC	CAGGAGGATGACCCTGTTTC	488
25 (g)	2,002	GGGTCATCCTCCTGTGTCTG	TAGCTAGCGCTTCTGGCATT	217

The *PARD3* gene exons (25) are listed along with the length of each exon in bp, the sequence of the forward and reverse primer used to amplify the *PARD3* genomic sequence corresponding to each exon (seven different primer sets were used to cover fully the very long (2,002 bp exon 25), and the length (bp) of the primer-amplified DNA fragment that was used for DNA sequencing.

Supplementary Table II. siRNAs targeting non-specific control and *PARD3*

Target RNA	Product name	Product number	Supplier
Non-targeting	ON-TARGETplus-Non-Targeting-Pool	D-001810-10	Dharmacon/GE Healthcare, Uppsala, Sweden
PARD3	ON-TARGETplus-Human-PARD3-siRNA-SMARTpool	L-015602-00-0020	Dharmacon/GE Healthcare, Uppsala, Sweden
PARD3	ON-TARGETplus-Human-PARD3-siRNA-#1	J-015602-05	Dharmacon/GE Healthcare, Uppsala, Sweden
PARD3	ON-TARGETplus-Human-PARD3-siRNA-#2	J-015602-06	Dharmacon/GE Healthcare, Uppsala, Sweden
PARD3	ON-TARGETplus-Human-PARD3-siRNA-#3	J-015602-07	Dharmacon/GE Healthcare, Uppsala, Sweden
PARD3	92 ON-TARGETplus-Human-PARD3-siRNA-#4	J-015602-08	Dharmacon/GE Healthcare, Uppsala, Sweden

Supplementary Table III. Antibodies and OPAL reagents

**Protein**	**Species**	**Dilution factor/Application**	**Supplier**
Caspase-3	rabbit	1:1,000/immunoblot	Cell Signaling Technology, Leiden, The Netherlands
GAPDH	mouse	1:15,000/immunoblot	Ambion, Thermo Fischer Scientific, Uppsala, Sweden
β-Actin	mouse	1:15,000/immunoblot	AC-15, Santa Cruz Biotechnology, Inc., Santa Cruz, CA, USA
PAR3	rabbit	1:500/immunoblot	Millipore/Merck, Stockholm, Sweden
PAR3	rabbit	1:100/IHC & PLA	Millipore/Merck, Stockholm, Sweden
PAR3, Opal 620 (FP1495001KT)		1:100/IHC	PerkinElmer Sverige AB, Upplands Väsby, Sweden
SOX2	rabbit	1:100/IHC	Abcam, Cambridge, UK
SOX2, Opal 540 (FP1494001KT)		1:100/IHC	PerkinElmer Sverige AB, Upplands Väsby, Sweden
Nestin	rabbit	1:100/IHC	Millipore/Merck, Stockholm, Sweden
Nestin, Opal 570 (FP1488001KT)		1:100/IHC	PerkinElmer Sverige AB, Upplands Väsby, Sweden
CD133	rabbit	1:50/IHC	Abcam, Cambridge, UK
CD133, Opal 480 (FP1500001KT)		1:100/IHC	PerkinElmer Sverige AB, Upplands Väsby, Sweden
GLAST-1	mouse	1:10/IHC	Miltenyi Biotec Norden AB, Lund, Sweden
GLAST-1, Opal 690 (FP1497001KT)		1:100/IHC	PerkinElmer Sverige AB, Upplands Väsby, Sweden
GFAP	rabbit	1:50/IHC	Santa Cruz Biotechnology Inc., Santa Cruz, CA, USA
GFAP, Opal 690 (FP1497001KT)		1:50/IHC	PerkinElmer Sverige AB, Upplands Väsby, Sweden
Ki67	rabbit	1:1,000/proliferation assay	Abcam, Cambridge, UK
anti-rabbit secondary Alexa-488	donkey	1:10,000/proliferation assay	Thermo Fischer Scientific, Uppsala, Sweden

Supplementary Table IV. RT-PCR primers

Gene	Forward primer (5´-3´)	Reverse primer (5´-3´)
hs*GAPDH*	GGAGTCAACGGATTTGGTCGTA	GGCAACAATATCCACTTTACCA
hs*PARD3*	TCCTCTTCCAGCTCCCATGA	CTGACGTCCAAATCCTTCTCGTT
hs*OLIG2*	CTTCAAGTCATCCTCGTCCAG	TGTTGATCTTGAGACGCAGC
hs*NES*	AGCCCTGACCACTCCAGTTTAG	CCCTCTATGGCTGTTTCTTTCTCT
hs*GFAP*	TGCGGCTCGATCAACTCA	GTTGGTTTCATCCTGGAGCTTCT
hs*CD133*	ACCCAACATCATCCCTGTTCTT	AGCTCTTCAAGGTGCTGTTCATG
hs*SOX2*	AGGGGGAAAGTAGTTTGCTGCCT	TGCCGCCGCCGATGATTGTT
hs*SSEA-1/FUT4*	GGTCCGCTACTACCACCAAC	CGAGTTCTCGAAAGCCAGGT
hs*βIIITUB*	CGCAGGACGCTCAAGGA	GCTCTTGGCACACACTTCCA
hs*MBP*	GCCCAGGGCACGCTTT	GCGTCTAGCCATGGGTGATC

Supplementary Table V

List of differentially expressed genes after Par3 silencing related to the data of Fig. 4. Excel table with 3 sheets labelled as follows and containing the following data:

3031_Par3_FDR_005: All differentially expressed genes with an FDR<0.05 comparing U3031MG cells transfected with siPar3 vs cells transfected with siControl.

3034_Par3_FDR_005: All differentially expressed genes with an FDR<0.05 comparing U3034MG cells transfected with siPar3 vs cells transfected with siControl.

Common genes: Genes down-regulated (logFC<-2) with an FDR<0.05 comparing U3031MG to U3034MG cells transfected with siPar3 vs cells transfected with siControl.

Supplementary figures

a

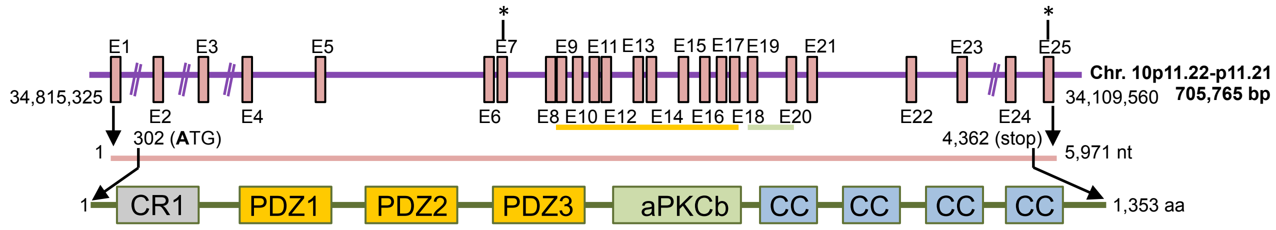

GBM cell	Unique SNPs				Common SNPs			
U3005MG	-		-		34,110,141 C→T, 95%	ex25 3'UTR	34,399,359 G→A, 99.9%	ex7 syn: His287
U3013MG	-		-		34,110,141 C→T, 95%	ex25 3'UTR	34,399,359 G→A, 99.9%	ex7 syn: His287
U3024MG	-		-		34,110,141 C→T, 95%	ex25 3'UTR	34,399,359 G→A, 99.9%	ex7 syn: His287
U3028MG	34,331,406 T→C, 7%	in18	-		34,110,141 C→T, 95%	ex25 3'UTR	34,399,359 G→A, 99.9%	ex7 syn: His287
U3031MG	-		-		34,110,141 C→T, 95%	ex25 3'UTR	34,399,359 G→A, 99.9%	ex7 syn: His287
U3034MG	34,269,911 G→A, 1.6%	in21	34,119,708 T→C, 22.6%	ex24 syn: R1191	34,110,141 C→T, 95%	ex25 3'UTR	34,399,359 G→A, 99.9%	ex7 syn: His287
U3062MG	34,331,292 C→T, 50%	ex19 syn: S886	34,360,218 G→C, 1.8%	ex25 syn: T1288	34,110,141 C→T, 95%	ex25 3'UTR	34,399,359 G→A, 99.9%	ex7 syn: His287

b

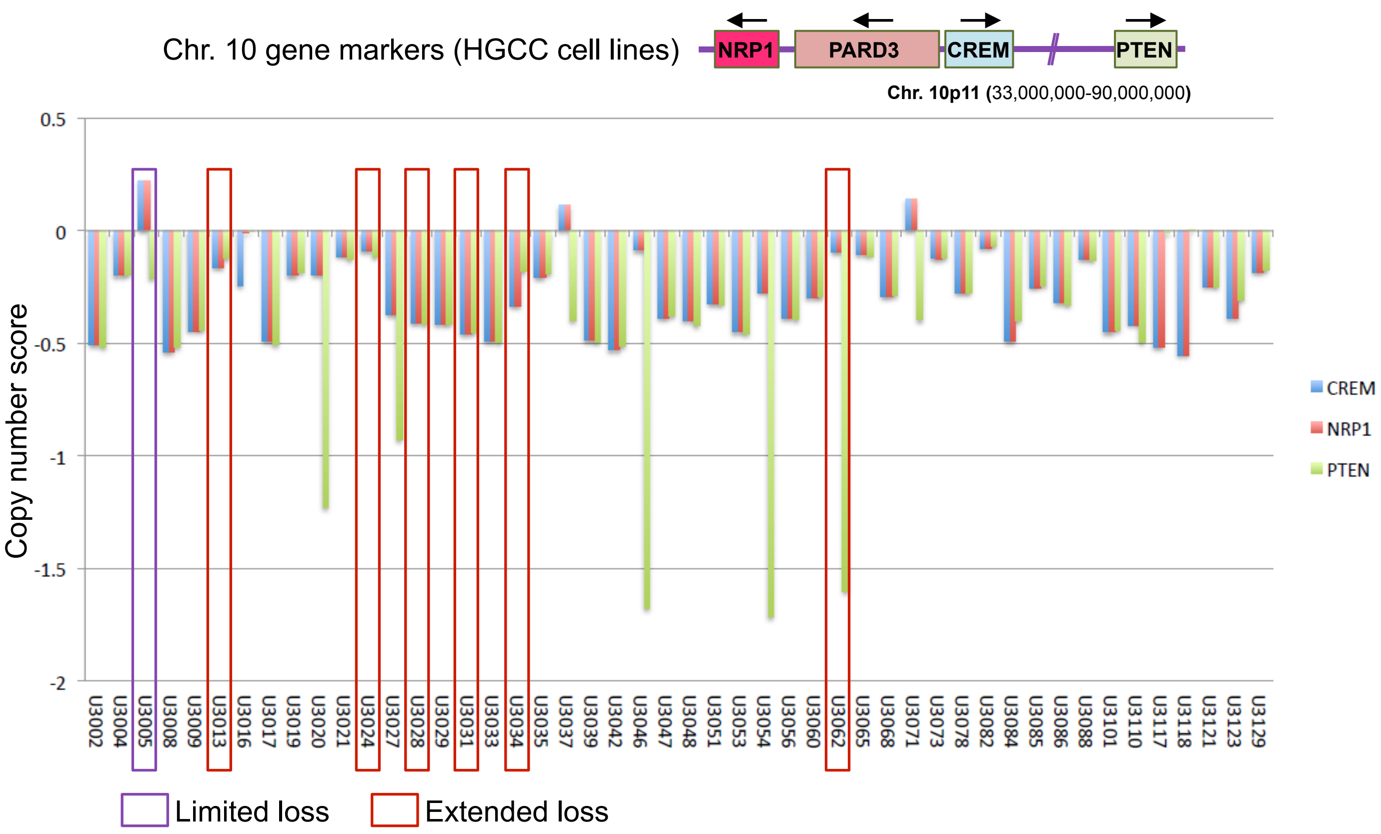

Supplementary Fig. S1

Fig. S1. *PARD3* genomic analysis in glioblastoma cells. a Graphic representation of the *PARD3* gene on chromosome 10p11.22-p11.21, spanning 705,765 bp (chromosomal bp coordinates are shown at each end of the gene). The 25 exons are drawn as boxes of equal length (out of scale) and the corresponding 24 introns are drawn as lines (in scale). Common SNPs are indicated with a star above the genomic locus representation. Colored underlines indicate exons encoding the protein domains marked in the protein diagram. Graphic representations of the *PARD3* mRNA isoform of 5,971 nt, marking the first nucleotide of the start and stop codons respectively, and of the PARD3/Par3 protein of 1,353 amino acids (aa), with characteristic protein domains (CR, conserved region; PDZ, PSD95/Dlg/ZO1 domain; aPKC, atypical protein kinase C domain; CC, coiled coil domain) depicted in color. The table lists the seven GBM lines with the corresponding SNPs identified after sequencing the *PARD3* exons and flanking intronic sequences. Common SNPs abundant in every sequenced culture are distinguished from SNPs identified only in few cultures (unique SNPs). Allelic frequency (%) of the base-pair identified in each culture along with its genomic location (in. intron; ex. exon; 3'UTR. 3' untranslated region), and impact on coding capacity (syn. synonymous) with corresponding amino acid and its coordinate in the Par3 protein sequence (superscript) are indicated. **b** Copy number score for 48 GBM cultures of the HGGC database, analyzed by probing for three genes in the *PARD3* locus, *CREM* (*cAMP responsive element modulator*), *NRP1* (*neuropilin 1*) and *PTEN* (*phosphatase and tensin homolog*). Diagram of chromosome 10 with the four relevant genes (colored as in the graph) and the orientation of their transcription (arrows). The seven GBM cultures analyzed in this paper are highlighted with boxes indicating limited (purple) or extended (red) genomic loss in chromosome 12 spanning the *PARD3* locus.

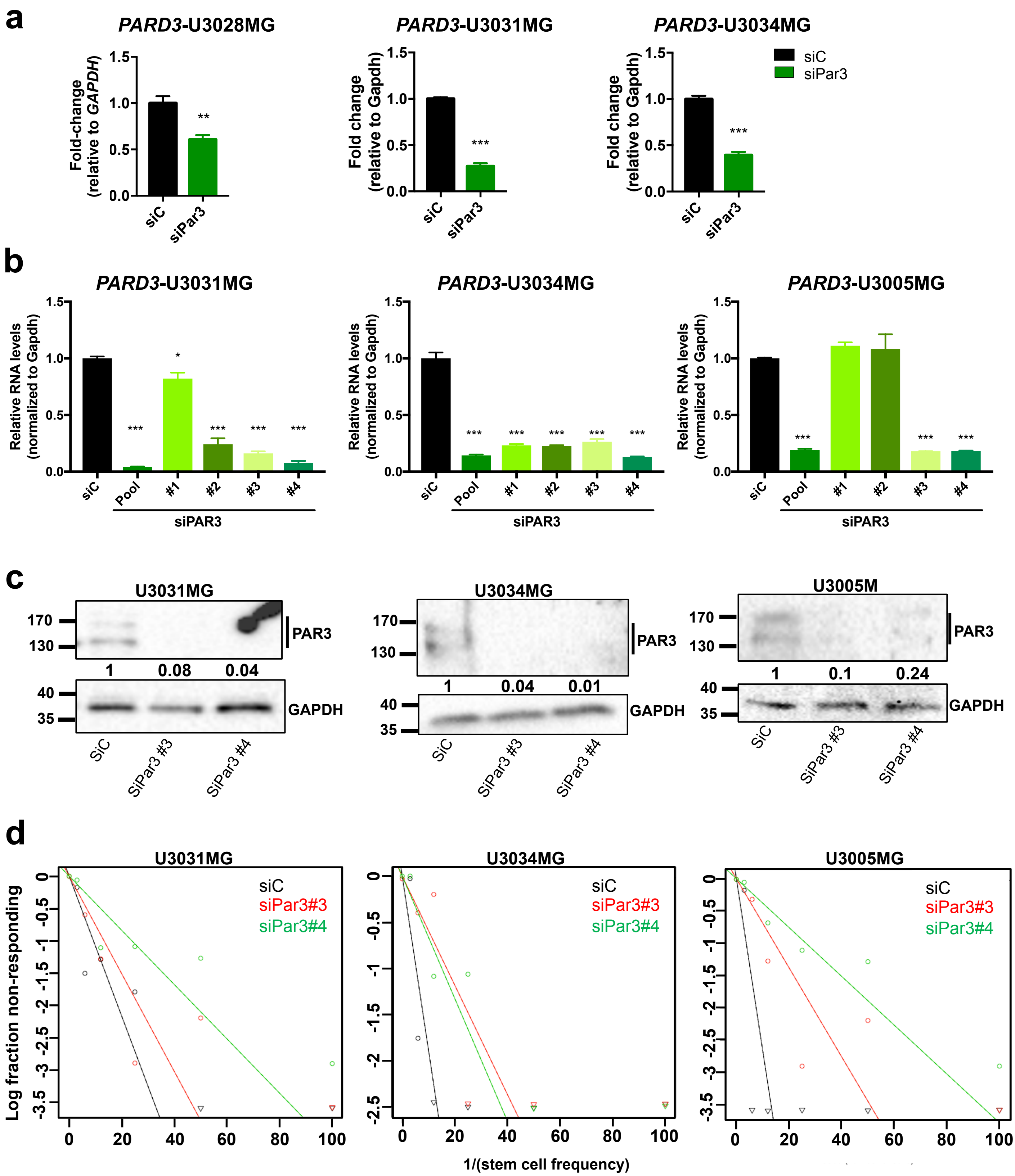

	U3031MG	U3034MG	U3005MG
siC	9.21	5.30	3.77
siPar3#3	13.22	16.90**	14.52***
siPar3#4	23.81***	15.05*	26.47***

Supplementary Fig. S2

Fig. S2. Par3 silencing inhibits gliomasphere formation. a Patient-derived GBM cultures were transiently transfected with siControl (siC, black bars) or siPar3 (pool, green bars) for 4 days. The expression of *PARD3* mRNA was measured in order to monitor the efficiency of Par3 silencing; results are mean±S.E.M. of n=2 (U3028MG) or n=5 (U3031MG and U3034MG) independent experiments with technical triplicates. Statistical comparison (t-test); significant differences, ***p*<0.01, ****p*<0.001. **b** Similar analysis as in panel (a) after transient transfection with siControl (siC, black bars) or siPar3 pool, #1, #2, #3 and #4 (green bars) for 3 days. Results are mean±S.E.M. of n=3 (U3031MG) or n=2 (U3034MG) and n=3 (U3005MG) independent experiments with technical triplicates. Statistical comparison (t-test); significant differences, **p<0.01, ***p<0.001. **c** Immunoblot of Par3, with GAPDH as loading control and densitometric values of Par3 relative to GAPDH listed, where the density of siC is normalized to 1. Molecular size markers in kDa are shown. **d** ELDA expressing median values from transfected cells (siControl (siC), black curves; siPar3#3, red curves; siPar3#4, green curves). High x-axis intercept corresponds to low number of gliomaspheres; note the large degree of shift of the median curves to the right upon Par3 silencing. The table shows the stem cell frequency (1 stem cell/x cells). For U3031MG, n=3 with 6 replicates; U3034MG, n=3 with 6 replicates; U3005MG, n=2 with 6 replicates.

Supplementary Fig. S3

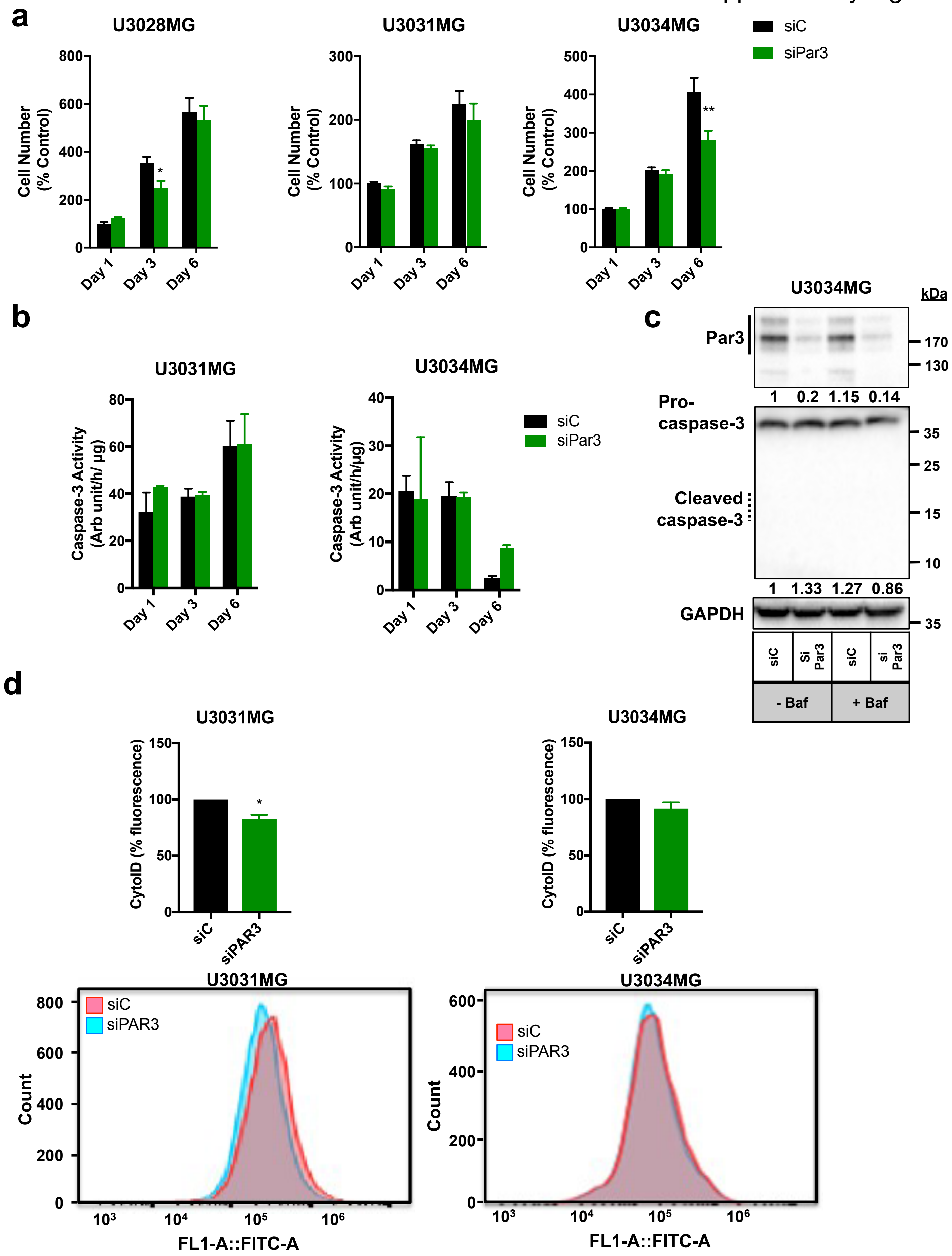

Fig. S3. Par3 silencing cannot induce apoptosis. Patient-derived GBM cultures were transiently transfected with siControl (siC, black bars) or siPar3 (green bars) for 4 days. **a** Cell viability was measured at the indicated times via MTS assay; results are mean±S.E.M of 3-5 independent experiments performed in triplicate. **b** Caspase-3 activity was measured at the indicated times; results are mean±S.E.M. of 2 independent experiments. **c** Immunoblot of Par3, intact and cleaved caspase-3, after 2 days of silencing and a final overnight treatment with bafilomycin (Baf, 40 nM), with GAPDH as loading control and molecular size markers in kDa shown. Densitometric values of Par3 relative to GAPDH are listed, where the density of siC is normalized to 1. **d** Autophagic vesicles were quantified by flow cytometry using Cyto-ID after two days of silencing and a final overnight treatment with chloroquine (40 μM). Left, results are mean±S.E.M. of n=4 independent experiments; right, representative histograms for each GBM culture.

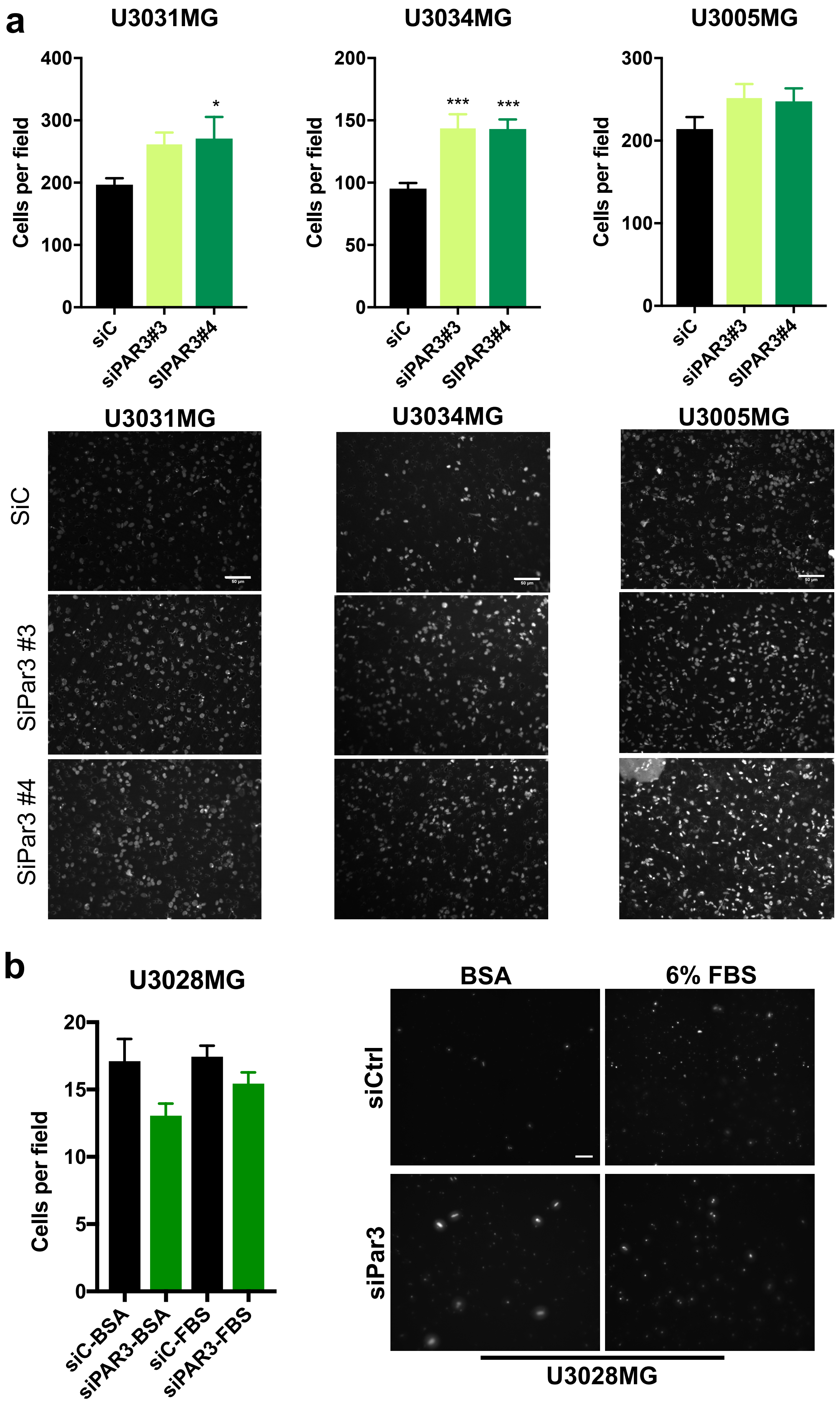

Supplementary Fig. S4

Fig. S4. Par3 silencing increases cell invasion. a Transwell-based invasion assays, of U3005MG, U3031MG and U3034MG cells transfected with control (black bars) or Par3#3 and #4 (green bars) siRNA, invading through laminin towards DMEM/6% FBS. The number of cells per field was quantified (for U3005MG, n=2; for U3031MG, n=2; for U3034MG, n=2; for each independent experiment 10 different fields were quantified). **b** Transwell-based invasion assays of U3028MG cells transfected with control (siC, black bars) or Par3 (green bars) siRNA, migrating through laminin towards DMEM/BSA or DMEM/6% FBS. Left, quantification of the number of cells per field (n=3, in duplicate each time; for each independent experiment 15 different fields were quantified). Right, representative photomicrographs of stained nuclei of invasive cells (magnification bar, 50 μm). Results are expressed as mean±SEM and statistical comparison (t-test) indicates significant differences, *$p<0.05$, ***$p<0.001$. Representative images of stained nuclei are shown below the graphs. Magnification bars; 50 μm.

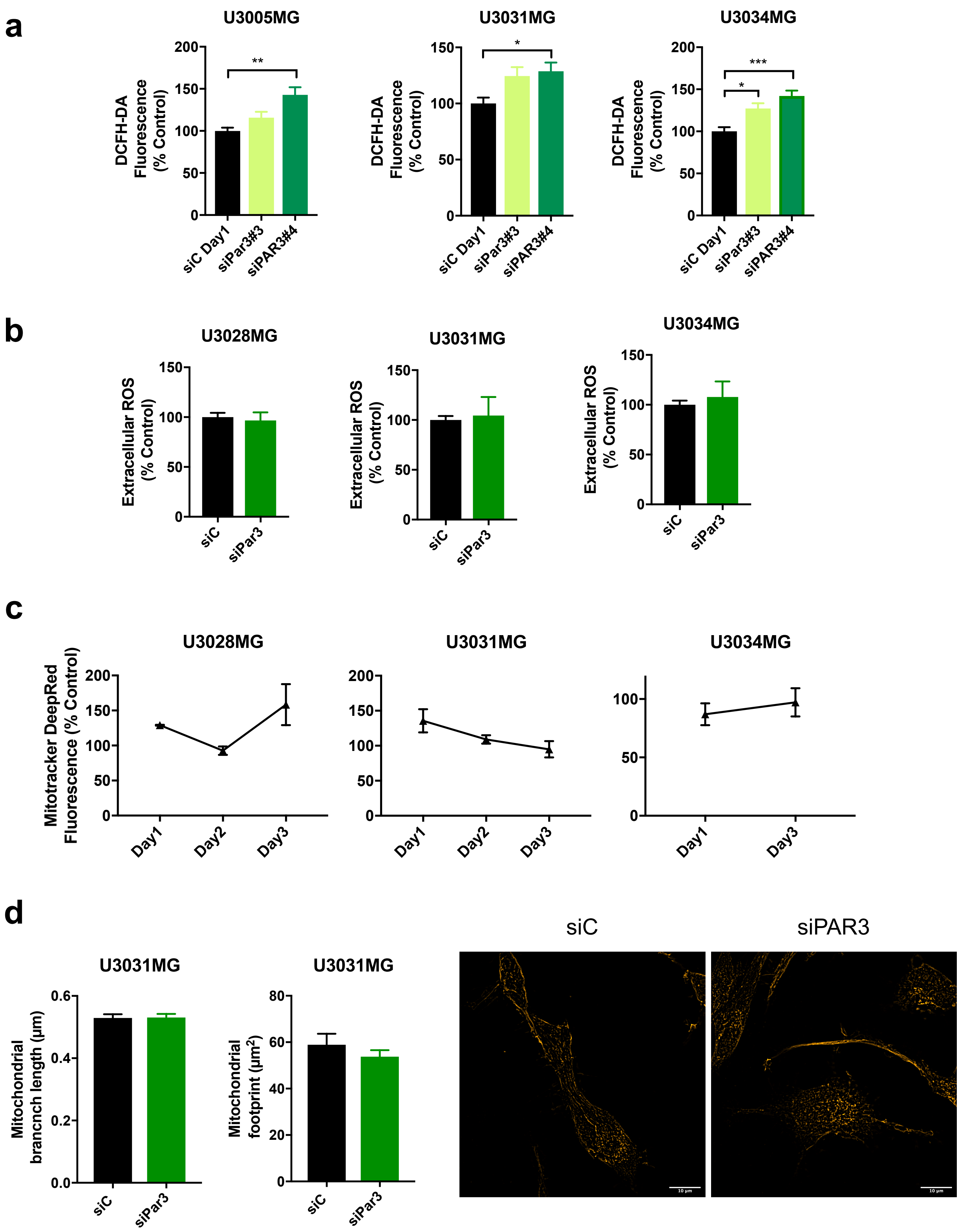

Supplementary Fig. S5

Fig. S5. Par3 silencing affects intracellular but not extracellular ROS production or mitochondrial mass and architecture. a U3005MG, U3031MG and U3034MG cells were transfected with control (black bars) and Par3#3 and #4 (green bars) siRNAs. Intracellular ROS content was measured by DCFH-DA fluorescence and expressed as percent of the control siRNA (siC). For U3005MG, n=2; for U3031MG, n=2; for U3034MG, n=2. **b** Extracellular ROS content was measured fluorimetrically using Amplex UltraRed after 3 days of transient transfection with control (black bars) and Par3 (green bars) siRNAs in the indicated GBM cultures. Results are mean±S.E.M. of 4 independent experiments performed in quadruplicate. **c** Analysis of mitochondrial mass by fluorimetry using MitoTracker Deep Red at the indicated time periods; results are mean±S.E.M. of 1-4 independent experiments. **d** Super-resolution confocal microscopic analysis of mitochondria stained with MitoTracker Deep Red in transiently transfected U3031MG cells for 3 days. Quantification of mitochondrial branch length and total mitochondrial surface area (footprint) is graphed and representative images are shown to the right (magnification bars; 10 μm). The results are mean±S.E.M. of 2-5 independent experiments.

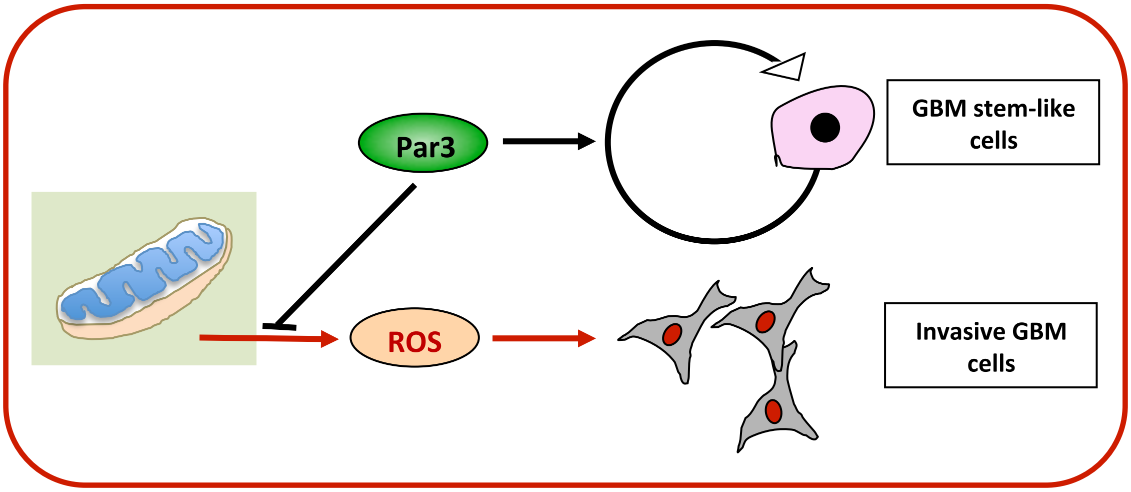

Supplementary Fig. S6

Fig. S6. Model of the dual role of Par3 in glioblastoma cells. Par3 positively contributes to the stem-like features of GBM cells (gliomasphere formation and enrichment in stem-like cells in GBM tissue). Par3 negatively regulates mitochondrial production of ROS that contribute to GBM cell invasiveness.
